# RIPK3 Orchestrates Scar‐Associated Macrophage Dysfunction to Drive Pulmonary Fibrosis

**DOI:** 10.1002/advs.202519404

**Published:** 2026-02-19

**Authors:** Tao Yang, Xiao Li, Shuyue Lei, Qingqing Li, Yi Zhang, Zhizhen Hui, Jinjin Ren, Xuelian Yang, Xiaoqian Yang, Chunlan Feng, Yousheng Xu, Dongxin Zhao, Wei Tang

**Affiliations:** ^1^ State Key Laboratory of Chemical Biology Shanghai Institute of Materia Medica Chinese Academy of Sciences Shanghai China; ^2^ School of Pharmacy University of Chinese Academy of Sciences Beijing China; ^3^ The Institute of Clinical Pharmacology Key Laboratory of Anti‐inflammatory and Immune Medicine Ministry of Education Anhui Collaborative Innovation Center of Anti‐inflammatory and Immune Medicine Anhui Medical University Hefei China; ^4^ School of Chinese Materia Medica Nanjing University of Chinese Medicine Nanjing China

**Keywords:** arginine metabolism, IPF, RIPK3, SAMs, SPP1

## Abstract

Idiopathic pulmonary fibrosis (IPF) is characterized by aberrant tissue remodeling and immune dysregulation. While receptor‐interacting protein kinase 3 (RIPK3) is canonically recognized as a central executioner of necroptosis, its non‐necroptotic functions in fibrosis remain unclear. Here, we identify a distinct, necroptosis‐independent immunometabolic function of RIPK3 in regulating pulmonary fibrosis. Significant upregulation of RIPK3 was found in IPF patients and mice and was particularly enriched in macrophages. Subsequently, macrophage‐specific RIPK3 knockout mice were established, which demonstrated resistance to bleomycin‐induced fibrosis. Single‐cell RNA sequencing further revealed that RIPK3 exerts its pro‐fibrotic effects by controlling the functional state of a specific subset of scar‐associated macrophages (SAMs). In vitro differentiation and functional analysis of SAMs from bone marrow‐derived monocytes confirmed *Spp1*, *Arg1*, and *Cx3cr1* as signature markers. Mechanistically, RIPK3 deficiency in SAMs inhibited the TGF‐β‐driven conversion of arginine to polyamines via the AKT‐mTOR pathway, thereby suppressing polyamine accumulation and its pro‐fibrotic effects. The translational potential of this finding was validated, as lung‐specific Ripk3 knockdown also attenuated lung fibrosis. Our findings extend RIPK3 biology beyond its classical role in cell death, highlighting RIPK3 as a key metabolic regulator of the fibrotic niche and suggesting that targeting this immunometabolic axis represents a promising therapeutic strategy for IPF.

## Graphical abstract

Beyond signaling cell death, RIPK3 emerges as a critical metabolic regulator in pulmonary fibrosis. This research reveals that RIPK3 promotes PI3K‐AKT signaling in scar‐associated macrophages to fuel polyamine synthesis, independent of its kinase activity. Targeting this unexpected immunometabolic axis effectively ameliorates fibrosis, offering a precise strategy to interrupt pro‐fibrotic cascades.

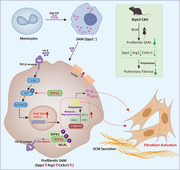

## 1 Introduction

Idiopathic pulmonary fibrosis (IPF) is a relentlessly progressive interstitial lung disease marked by declining pulmonary function and excessive extracellular matrix deposition, leading to a median survival of only 2–4 years [1]. Although the antifibrotic agents pirfenidone and nintedanib could slow disease progression, their effects on overall survival are modest and are frequently accompanied by considerable adverse events. Notably, a novel PDE4B inhibitor, Nerandomilast, achieved its primary endpoint in a Phase III trial and subsequently entered the new drug application process in 2025 [2]. This development constitutes the first significant breakthrough in Phase III IPF therapy in over a decade, with its benefits stemming from both anti‐inflammatory and antifibrotic actions [3, 4]. The pathogenesis of IPF involves recurrent alveolar epithelial injury coupled with aberrant repair mechanisms. Continuous epithelial damage elicits a maladaptive inflammatory response, and the ensuing imbalance between inflammatory and fibrotic processes ultimately results in irreversible lung remodeling [5, 6, 7]. Furthermore, the dysregulated activation of various cytokines and signaling pathways within the immune microenvironment is believed to be pivotal in both initiating and driving IPF progression [8].

Receptor‐interacting protein kinase 3 (RIPK3), a central mediator of necroptosis, has long been recognized for its role in the RIPK1‐RIPK3‐mixed lineage kinase domain like (MLKL) cell death cascade [9, 10]. However, emerging evidence indicates that RIPK3 possesses multifaceted regulatory capabilities that extend beyond its lethal function. In non‐lethal contexts, RIPK3 modulates a range of biological processes, including NF‐κB signaling [11, 12, 13, 14], inflammasome activation [15, 16, 17], autophagy [18, 19, 20], and cellular metabolism [21, 22, 23], via both kinase‐dependent and kinase‐independent mechanisms. Importantly, RIPK3 has been implicated in the pathogenesis of several fibrotic diseases, such as nonalcoholic fatty liver disease [24, 25, 26, 27, 28, 29, 30, 31], lung fibrosis [32, 33, 34], and renal fibrosis [35, 36, 37, 38, 39, 40, 41, 42]. While current research on lung fibrosis has largely centered on RIPK3‐driven necroptosis in parenchymal cells, the specific cell populations affected by RIPK3 within the fibrotic lung microenvironment and the intracellular pathways involved remain insufficiently characterized.

Macrophages, which are abundant in lung tissue, are integral to maintaining pulmonary homeostasis and orchestrating immune defense. Throughout IPF, lung macrophages undergo complex phenotypic transitions, influencing inflammatory responses, tissue repair, and aberrant fibrotic processes. Traditionally, alveolar macrophages (AMs) have been considered the predominant tissue‐resident population, residing within the alveolar spaces [43, 44]. In contrast, interstitial macrophages (IMs), located in the lung parenchyma, particularly near blood vessels, major airways, and the alveolar interstitium, represent a distinct subset [45, 46]. Although the M1/M2 polarization model is commonly used to delineate pro‐inflammatory versus anti‐inflammatory functions, this dichotomy fails to capture the extensive heterogeneity of macrophage phenotypes present in the fibrotic milieu. Recent single‐cell transcriptomic analyses have identified multiple macrophage subpopulations with unique functional profiles. For instance, scar‐associated macrophages (SAMs), characterized by high expression of *Spp1*, *Gpnmb*, *Trem2*, *Cd9*, *Fabp5*, and *Cd63*, are closely associated with fibrotic pathology [47, 48, 49]; Ly6G^+^ macrophages facilitate epithelial regeneration after viral infections [50]; and platelet‐instructed SPP1^+^ macrophages, distinguished by elevated *Spp1*, *Fn1*, and *Arg1* expression, have been linked to heart failure and chronic kidney disease [51]. Nonetheless, a systematic exploration of the phenotypic traits and regulatory mechanisms governing key macrophage subsets in lung fibrosis is still lacking.

To address this, we integrated clinical data analysis, conditional gene knockout mouse models, single‐cell sequencing, and metabolic profiling to systematically dissect the regulatory role of RIPK3 in SAMs and its impact on lung fibrosis progression. Our findings demonstrated for the first time that RIPK3 regulates arginine metabolism in SAMs, promoting polyamine synthesis and driving pulmonary fibrosis. This study uncovers a distinct necroptosis‐independent immunometabolic axis, offering a theoretical foundation for targeting macrophage metabolic reprogramming as a therapeutic strategy for fibrotic diseases.

## 2 Results

### 2.1 Upregulated RIPK3 Expression in Patients With IPF and Mouse Models

**FIGURE 1:**
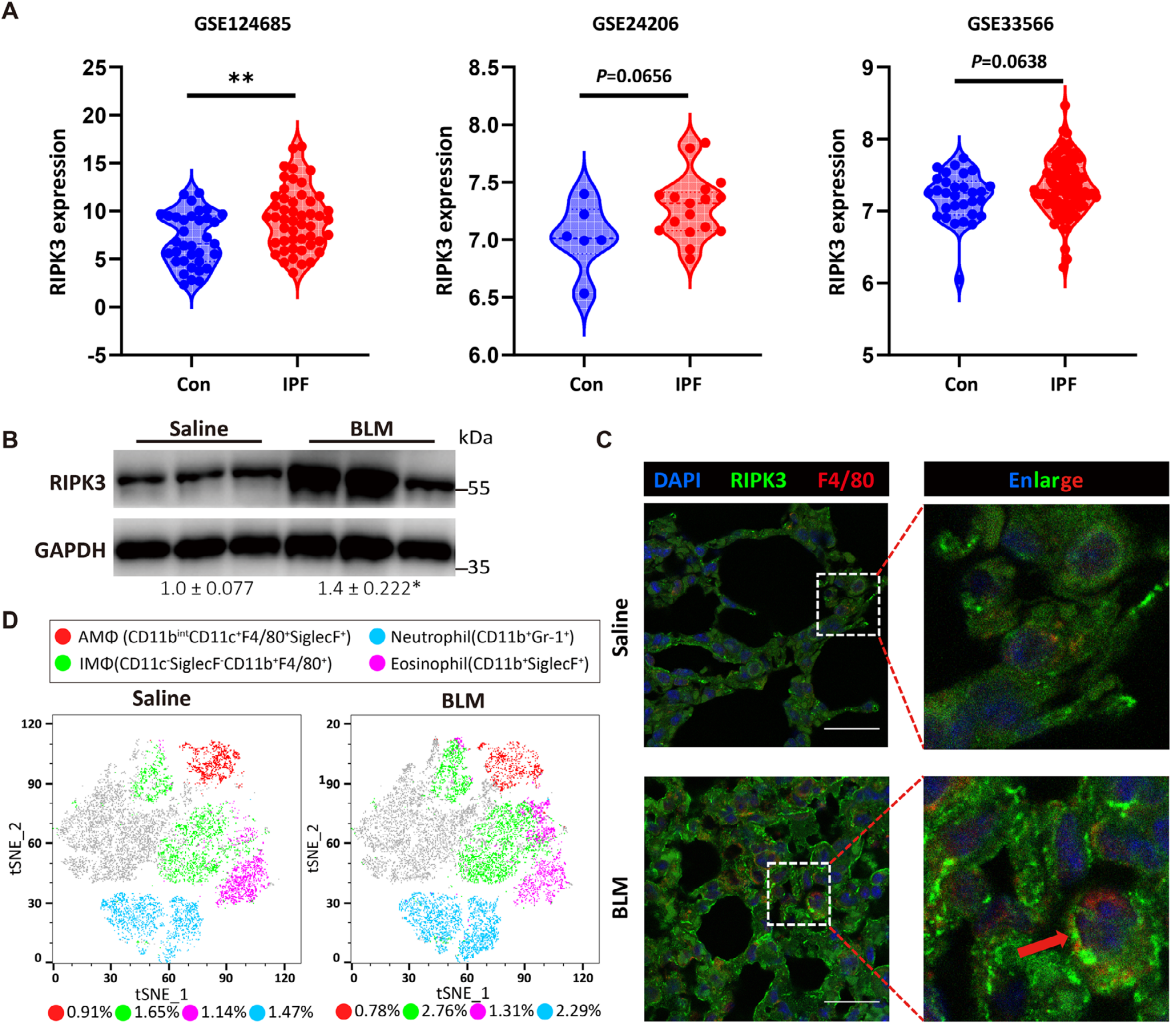
RIPK3 increase in patients with IPF and a mouse model of BLM‐induced pulmonary fibrosis. (A) Expression of *RIPK3* in lung tissues from IPF patients (GSE124685 (n_Con_ = 35, n_IPF_ = 49)), GSE24206 (n_Con_ = 7, n_IPF_ = 17) and peripheral blood (GSE33566 (n_Con_ = 30, n_IPF_ = 93)) from public GEO databases. (B) Protein expression levels of RIPK3 in lung tissues from BLM‐induced pulmonary fibrosis in mice, *n* = 3. (C) Representative immunofluorescence images of RIPK3 and F4/80 in lung tissue.(D) Representative t‐SNE flow cytometry analysis plots of CD11b^int/+^ cells in lung tissue and the proportion of these cells among total lung single cells. Scale bar: 20 µm. (A,B) Unpaired two‐tailed Student's *t*‐tests were used. ^*^ *p* < 0.05, ^**^ *p* < 0.01 compared with the Saline group.

Despite previous studies suggesting that RIPK3 may play a critical role in pulmonary fibrosis, its specific pathological mechanisms remain incompletely understood. To systematically investigate the pathophysiological role of RIPK3 in pulmonary fibrosis, we first conducted bioinformatics analysis using publicly available data from the GEO database to examine *RIPK3* expression profiles in patients with IPF. The results revealed that *RIPK3* mRNA levels were upregulated in both IPF patient lung tissues and peripheral blood compared to healthy controls (Figure 1A). In a bleomycin (BLM)‐induced pulmonary fibrosis mouse model, we observed that RIPK3 protein expression was significantly elevated in fibrotic lung tissues (Figure 1B). Based on annotations from the Human Protein Atlas database (https://www.proteinatlas.org/), RIPK3 is primarily localized to parenchymal cells, such as alveolar epithelial and endothelial cells in lung tissue. In immune cells, its expression is predominantly restricted to macrophages. Immunofluorescence staining revealed that overall RIPK3 expression was significantly elevated in lung tissues, with high levels observed in macrophages (Figure 1C; Figure S2A). To definitively confirm this cell‐type‐specific upregulation in vivo, we isolated primary lung macrophages from saline‐ and BLM‐treated mice using anti‐F4/80 immunomagnetic sorting. Both RT‐qPCR and Western blot analyses demonstrated a robust induction of RIPK3 expression specifically within the macrophage fraction of fibrotic lungs compared to controls (Figure S2B,C). To further explore the role of macrophage subsets in pulmonary fibrosis, we performed t‐distributed stochastic neighbor embedding (t‐SNE) analysis on CD11b^int/+^ cells isolated from mouse lung tissues. The results demonstrated that while the proportion of alveolar macrophages (AMΦ) remained relatively stable under fibrotic conditions, interstitial macrophages (IMΦ) were expanded (Figure 1D). This suggests that macrophages may serve as a critical target cell type for RIPK3‐mediated pathological effects.

### 2.2 RIPK3 Ablation in Macrophages Mitigates BLM‐Induced Lung Injury

**FIGURE 2:**
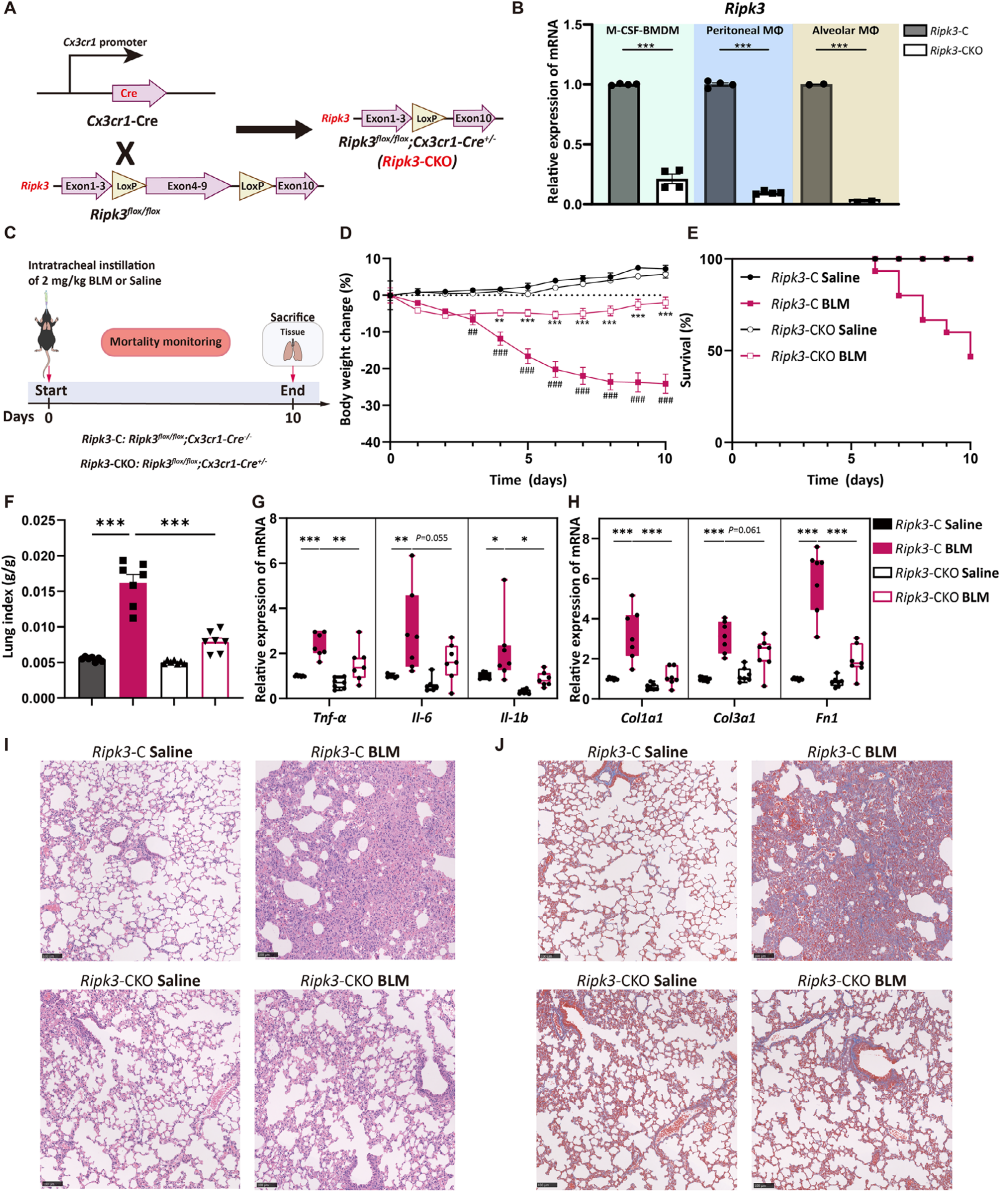
RIPK3 deletion in macrophages attenuates BLM‐induced pulmonary injury. (A) Schematic diagram illustrating the construction of macrophage‐specific RIPK3 knockout (*Ripk3*‐CKO) mice. (B) RT‐qPCR analysis of *Ripk3* gene expression levels in BMDMs, peritoneal macrophages, and alveolar macrophages from *Ripk3*‐C and *Ripk3*‐CKO mice. (C) Schematic diagram of the BLM‐induced lung injury model construction (*n* = 7 to 14 mice per group). (D) Body weight changes in mice with BLM‐induced lung injury. (E) Survival rate of mice with BLM‐induced lung injury. (F) Lung index of mice with BLM‐induced lung injury. (G) Gene expression levels of inflammatory cytokines *TNF‐α*, *IL‐6*, and *IL‐12* in mouse lung tissues were detected by RT‐qPCR. (H) Gene expression levels of *Col1a1*, *Col3a1*, and *Fn1* in mouse lung tissues were detected by RT‐qPCR. (I,J) Representative images of H&E staining and Masson's trichrome staining of lung tissue. Scale bar, 100 µm. [(B), (D), (F‐H)] Data show means ± SEM and are pooled from at least two independent experiments. Symbols on bar graphs represent individual mice. (B) unpaired two‐tailed Student's *t*‐test, (D) two‐way ANOVA with Dunnett's multiple comparisons test, (F–H) one‐way ANOVA with Dunnett's multiple comparisons test were used. ^##^ *p* < 0.01, ^###^ *p* < 0.001, compared with the *Ripk3*‐C Saline group, ^*^ *p* < 0.05, ^**^ *p* < 0.01, ^***^ *p* < 0.001 compared with the *Ripk3*‐C BLM group or indicated groups.

To further elucidate the specific function of RIPK3 in macrophages, we generated a mouse model with macrophage‐selective deletion of RIPK3. This model was generated by crossing *Ripk3^flox/flox^* mice with *Cx3cr1*‐*Cre* mice. *Ripk3^flox/flox^; Cx3cr1‐Cre^+/−^* mice (designated as *Ripk3*‐CKO) served as the experimental group, while their littermate *Ripk3^flox/flox^*; *Cx3cr1*‐*Cre* ^−/−^ mice (designated as *Ripk3*‐C) were used as controls (Figure 2A; Figure S2D,E). The deletion of RIPK3 was confirmed in bone marrow‐derived macrophages (BMDMs), peritoneal macrophages (PMs), and alveolar macrophages (AMs) (Figure 2B; Figure S2F). To determine whether RIPK3 plays a pathological role during the early stages of lung injury, we induced lung injury by intratracheal instillation of BLM at a dose of 2 mg/kg (Figure 2C). This dosage causes relatively severe lung tissue damage, allowing for a better simulation of the pathological changes observed in the early stages of pulmonary fibrosis. Results showed that compared to the *Ripk3*‐C BLM group, *Ripk3*‐CKO BLM mice exhibited significantly less weight loss (Figure 2D) and a significantly higher survival rate (Figure 2E). Furthermore, the lung index was significantly lower in *Ripk3*‐CKO BLM mice compared to the control group (Figure 2F). The mRNA expression levels of inflammatory cytokines (*Tnf‐α*, *Il‐6*, and *Il‐12*) and fibrosis‐related genes (*Col1a1*, *Col3a1*, and *Fn1*) were significantly decreased in the lung tissues of *Ripk3*‐CKO BLM mice (Figure 2G,H). H&E and Masson's trichrome staining showed that the lungs of *Ripk3*‐C BLM mice displayed typical pathological features of fibrosis, including thickened alveolar septa, extensive immune cell infiltration, destruction of bronchial structures, alveolar collapse, and increased collagen deposition. In contrast, *Ripk3*‐CKO mice exhibited significantly attenuated lung tissue damage and markedly reduced areas of collagen deposition (Figure 2I,J).

### 2.3 RIPK3 Ablation in Macrophages Mitigates BLM‐Induced Lung Fibrosis

**FIGURE 3:**
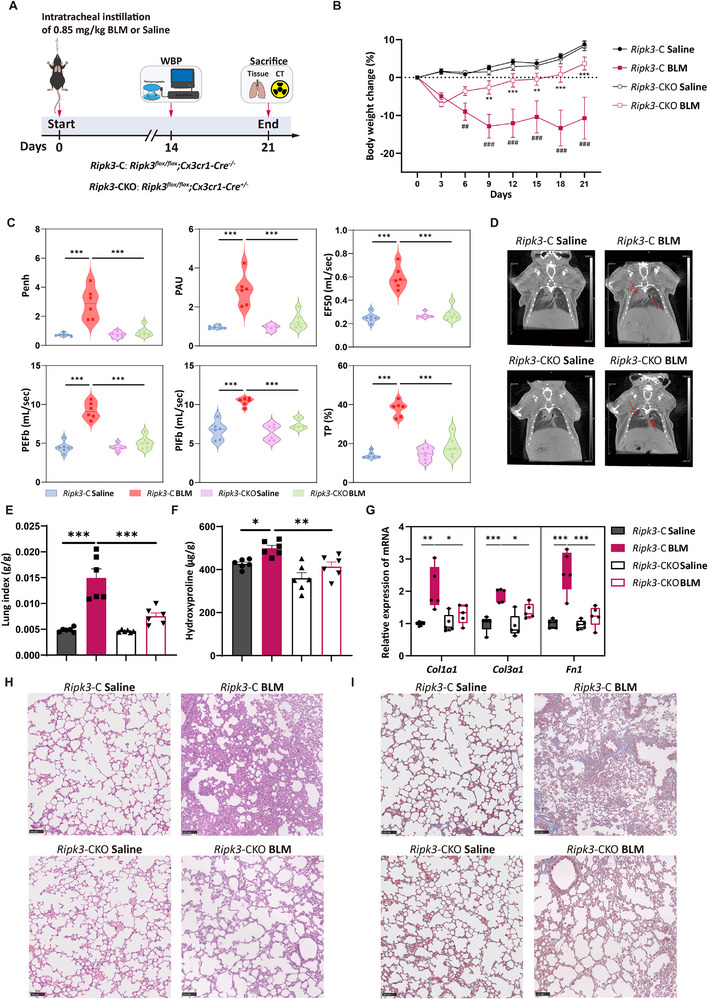
RIPK3 deletion in macrophages attenuates BLM‐induced pulmonary fibrosis. (A) Schematic diagram of the BLM‐induced lung fibrosis model construction (*n* = 5 to 6 mice per group). (B) Body weight changes in mice with BLM‐induced pulmonary fibrosis. (C) Respiratory parameters related to pulmonary fibrosis detected by Whole Body Plethysmography (WBP): enhanced pause (Penh), pause (PAU), peak expiratory flow (PEFb), peak inspiratory flow (PIFb), mid‐expiratory flow (EF50), and pause time ratio (TP). (D) Representative Micro‐CT images of the lungs. Red arrows in the images indicate areas of differential lung density. (E) Lung index of mice with BLM‐induced pulmonary fibrosis. (F) Hydroxyproline content in lung tissue from mice with BLM‐induced pulmonary fibrosis. (G) Gene expression levels of *Col1a1*, *Col3a1*, and *Fn1* in mouse lungs detected by RT‐qPCR. (H,I) Representative images of H&E staining and Masson's trichrome staining of lung tissue pathological sections (scale bar: 100 µm). [(B), (E–G)] Data show means ± SEM. Symbols on bar graphs represent individual mice. (B) Two‐way ANOVA with Dunnett's multiple comparisons test, [(C), (E–G)] one‐way ANOVA with Dunnett's multiple comparisons test were used. ^##^ *p* < 0.01, ^###^ *p* < 0.001, compared with the *Ripk3*‐C Saline group, ^*^ *p* < 0.05, ^**^ *p* < 0.01, ^***^ *p* < 0.001 compared with the *Ripk3*‐C BLM group or indicated groups.

Building upon our previous findings that macrophage‐specific deletion of RIPK3 alleviated lung injury, we further investigated its impact on the development of chronic pulmonary fibrosis. To mimic the progressive pathological features observed in clinical pulmonary fibrosis, we established a chronic pulmonary fibrosis model using intratracheal instillation of BLM at a lower dose of 0.85 mg/kg (Figure 3A). The results showed that *Ripk3*‐CKO BLM mice exhibited significantly less body weight loss post‐induction compared to the *Ripk3*‐C BLM mice (Figure 3B). Furthermore, key respiratory parameters measured by WBP indicated significantly improved respiratory function in *Ripk3*‐CKO mice, evidenced by the normalization trends in enhanced pause (Penh), pause (PAU), peak expiratory flow (PEFb), peak inspiratory flow (PIFb), expiratory flow at 50% volume (EF50), and tidal volume (TV) (Figure 3C). Moreover, Micro‐CT analysis revealed significantly reduced high‐density regions (indicative of fibrotic lesions and inflammatory infiltration) in the lungs of *Ripk3*‐CKO BLM mice (Figure 3D). The lung index and hydroxyproline content showed a significant reduction in the lung tissue of *Ripk3*‐CKO mice compared to controls (Figure 3E,F). The mRNA expression levels of fibrosis‐related genes (*Col1a1*, *Col3a1*, and *Fn1*) were significantly downregulated in the lung tissues of *Ripk3*‐CKO mice compared to controls (Figure 3G). Histopathological analysis corroborated these findings, with H&E and Masson's trichrome staining showing significantly attenuated lung tissue damage and markedly reduced areas of collagen deposition in *Ripk3*‐CKO mice (Figure 3H,I).

To further validate the pathological role of RIPK3 in pulmonary fibrosis, we generated a mouse model with macrophage‐selective overexpression of RIPK3. This model was generated by crossing *Rosa26^stop(flox/flox)Ripk3^* mice with *Cx3cr1‐Cre* mice. The resulting *Rosa26^stop(flox/+)Ripk3^*; *Cx3cr1*‐*Cre^+/−^* mice (designated *Ripk3‐*CKI) served as the experimental group, while their littermate *Rosa26^stop(flox/flox)Ripk3^*; *Cx3cr1*‐*Cre^−/−^* mice (designated *Ripk3*‐C) were used as controls (Figure S3A,B). However, we observed that *Ripk3*‐CKI mice exhibited gradual growth retardation after birth and increased progressive mortality between 4 and 6 weeks, suggesting that sustained overexpression of RIPK3 may induce systemic pathological effects. Given the developmental abnormalities observed in *Ripk3*‐CKI mice, we further investigated changes within their pulmonary immune microenvironment. Flow cytometry analysis of lung tissues revealed significant infiltration of myeloid cells (CD45^+^CD11b^+^) in *Ripk3*‐CKI mice. This was characterized primarily by a surge in neutrophils (CD11b^+^Gr1^+^) and an increase in macrophage numbers (CD11b^+^F4/80^+^), while the number of alveolar macrophages (CD11b^int/+^SiglecF^+^) remained unchanged (Figure S3C). Also, we performed flow cytometric analysis on other important organs such as the liver, spleen, skin, and colon, and found that they were also similarly infiltrated by large numbers of neutrophils (data not shown). These results from reverse genetic studies demonstrate that overexpression of RIPK3 in macrophages disrupts immunological homeostasis and leads to systemic fatal inflammation, suggesting that RIPK3 may play a critical role in the early inflammatory response in pulmonary injury and fibrosis.

### 2.4 RIPK3 Deficiency Blunts the Pro‐Fibrotic Phenotype of Lung Macrophages

Based on the traditional macrophage polarization theory, we performed in vitro experiments to assess the impact of RIPK3 on macrophage polarization phenotypes. Experimental results indicated that in RIPK3‐deficient BMDMs generated with M‐CSF, there were no significant differences in the expression of pro‐inflammatory cytokines or canonical M1 and M2 polarization markers under either M1 or M2 polarizing conditions (Figure S4). These findings, seemingly contradictory to the traditional polarization theory, suggest that the regulation of macrophage function by RIPK3 cannot be fully captured by the classical M1/M2 dichotomy.

**FIGURE 4:**
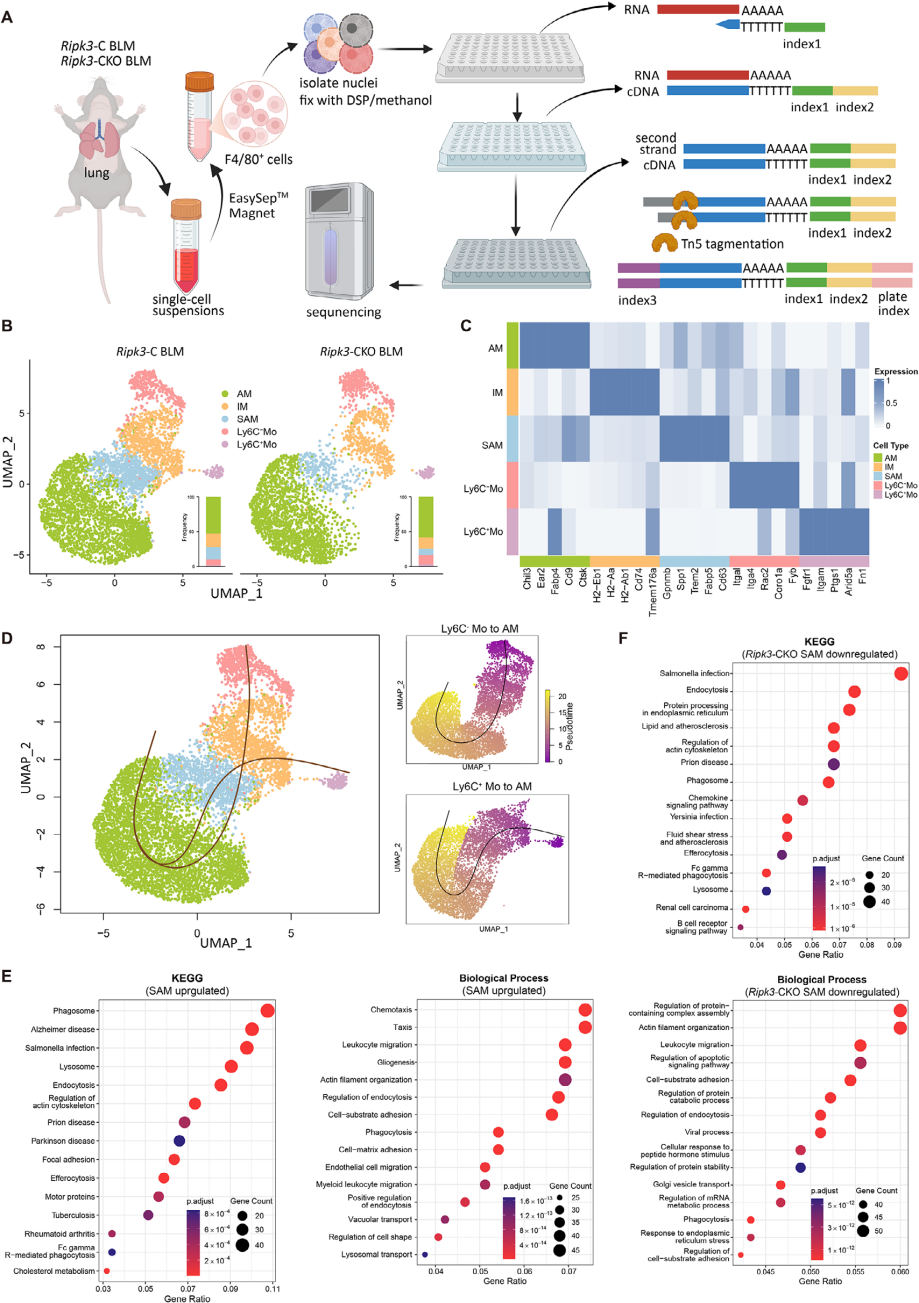
RIPK3 deficiency reduced the proportion of SAMs in fibrotic lung tissue. (A) Schematic diagram of the single‐cell combinatorial indexing RNA sequencing (sci‐RNA‐seq) workflow for pulmonary macrophages in pulmonary fibrosis. The schematic diagram was created in biorender.com. (B) UMAP plots of mononuclear phagocytes from cell clusters sorted by F4/80 magnetic beads labeled by 5 cell types. (C) Heatmap showing the expression of major differentially expressed genes (DEGs) among different subpopulations. (D) UMAP plot depicting the transcriptional identity and cell trajectories (left) and pseudotime trajectory values (right) of lung AMs, IMs, SAMs, Ly6C^−^ Mos, and Ly6C^+^ Mos, as in (B), evaluated by Slingshot trajectory analyses. (E) GSEA of the SAM profile compared with other clusters using the Kyoto Encyclopedia of Genes and Genomes (KEGG) and Gene Ontology (GO) biological process gene sets. The normalized enrichment score, p.adjust, and gene count are shown for each process. (F) GSEA of the SAM profile in *Ripk3*‐C and *Ripk3*‐CKO groups using the KEGG and GO biological process gene sets. The normalized enrichment score, p.adjust, and gene count were shown for each process.

Recent single‐cell transcriptomic studies have revealed highly heterogeneous macrophage subpopulations during pulmonary fibrosis progression, whose functional characteristics often deviate significantly from simple M1/M2 labels, highlighting the limited explanatory power of the traditional polarization model in complex pathological contexts [47, 48, 49, 50, 51, 52, 53, 54, 55, 56, 57]. Therefore, a more detailed functional classification of macrophages is required to investigate how RIPK3 modulates macrophage involvement in pulmonary fibrosis. To systematically resolve the macrophage heterogeneity regulated by RIPK3 during pulmonary fibrosis, we performed single‐cell combinatorial indexing RNA sequencing (sci‐RNA‐seq) on lung tissues from BLM‐treated *Ripk3*‐CKO and *Ripk3*‐C mice. Macrophages were efficiently enriched from lung single‐cell suspensions using F4/80 magnetic bead sorting. Subsequently, macrophage nuclei were isolated, and RNA‐seq libraries were constructed using Split‐pool barcoding, followed by high‐throughput sequencing (Figure 4A). Following dimensionality reduction using Principal Component Analysis (PCA) and Uniform Manifold Approximation and Projection (UMAP), clustering analysis partitioned the mononuclear phagocytes into five distinct subpopulations (Figure 4B; Figure S5A–D). These were identified based on their unique gene signatures as alveolar macrophages (AMs; e.g., *Chil3*, *Ear2*), interstitial macrophages (IMs; e.g., *H2‐Aa*, *Cd74*), scar‐associated macrophages (SAMs; e.g., *Gpnmb*, *Spp1*), Ly6C^−^ monocytes (Ly6C^−^Mos; e.g., *Itga1*, *Itga4*), and Ly6C^+^ monocytes (Ly6C^+^Mos; e.g., *Itgam*, *Fgfr1*) (Figure 4C; Figure S5E,F).

To delineate the differentiation dynamics of mononuclear phagocytes in IPF, we performed pseudotime trajectory analysis. This revealed a convergent differentiation axis where trajectories originating from both Ly6C^−^ and Ly6C^+^ monocytes culminated in AM fate (Figure 4D). Both pathways were channeled through an intermediate hub of SAMs, a cell population previously implicated in the progression of fibrosis [47, 48, 49, 50, 51, 52, 53, 55]. The transient identity of this hub was confirmed by its gene expression dynamics; key SAM signature genes, such as *Spp1*, *Mmp14*, *Cd9*, and the *C1q* complement cascade, were transiently expressed, peaking exclusively at the SAM nexus (Figure S5H). These findings indicate SAMs as an essential cellular hub in the IPF lung, acting as an obligate checkpoint that channels heterogeneous monocyte precursors toward a unified AM identity. Gene set enrichment analysis (GSEA) identified SAMs as a distinct, immune‐activated cell population characterized by strong migratory and phagocytic capabilities (Figure 4E). The importance of RIPK3 to this population was underscored by a significant decrease in their proportion and a lower odds ratio of cells adopting the SAM phenotype in *Ripk3*‐CKO mice (Figure 4B; Figure S5G). To confirm its role as a driver, we analyzed downregulated pathways upon Ripk3 knockout and found a stark reversal of the SAM phenotype, with significant suppression of hallmark pathways governing both phagocytosis and cell motility (Figure 4F). Therefore, these results suggest that RIPK3 is an important regulator that contributes to the establishment and maintenance of the activated, migratory, and phagocytic phenotype of SAMs.

### 2.5 RIPK3 Ablation Inhibits SAMs Function

**FIGURE 5:**
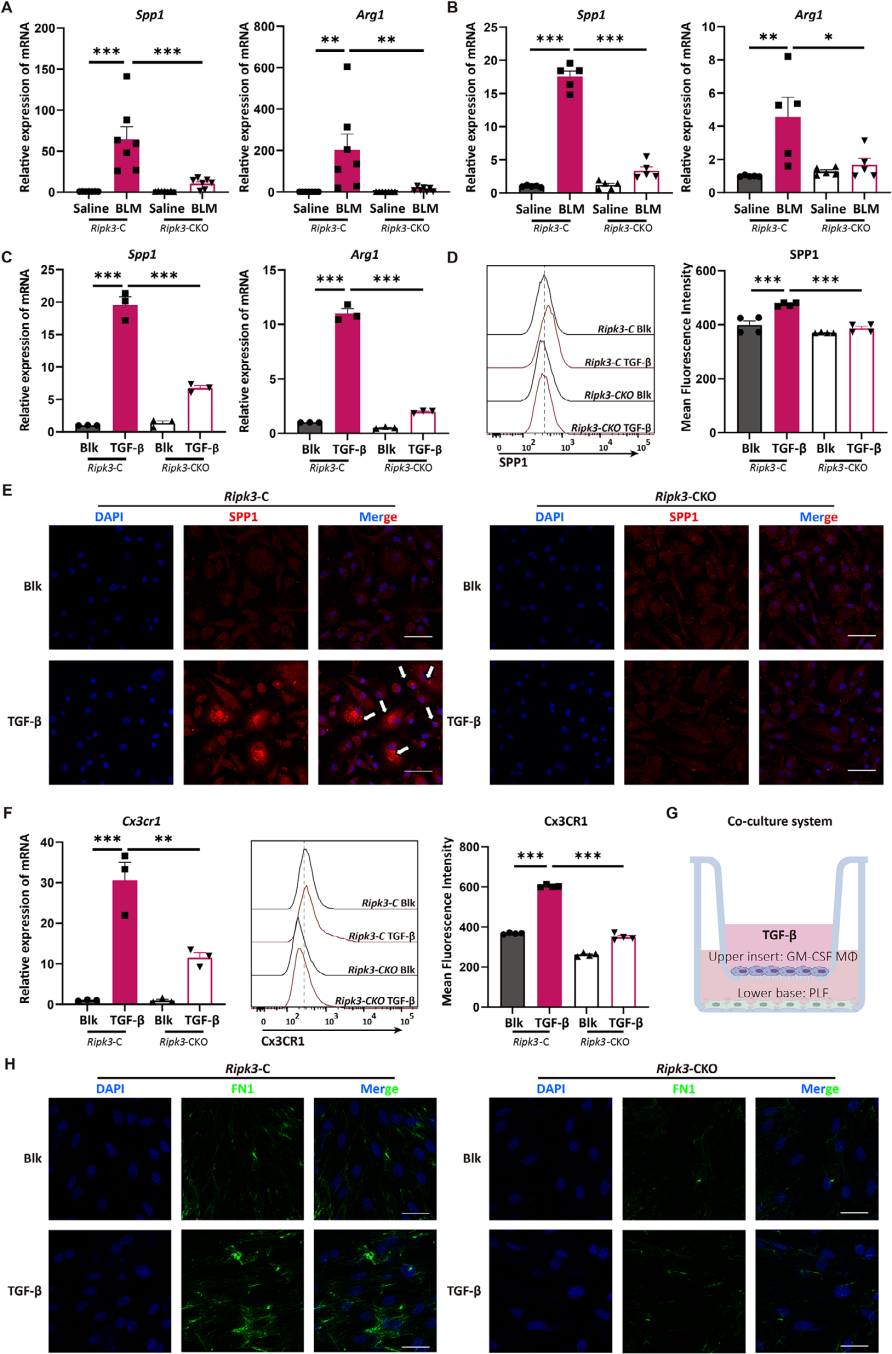
Deletion of RIPK3 suppressed the pro‐fibrotic phenotype of SAMs. (A) Gene expression of *Spp1* and *Arg1* in lung tissue from mice with 2 mg/kg BLM‐induced lung injury, detected by RT‐qPCR. (B) Gene expression of *Spp1* and *Arg1* in lung tissue from mice with 0.85 mg/kg BLM‐induced pulmonary fibrosis, detected by RT‐qPCR. (C) Gene expression of *Spp1* and *Arg1* in SAMs, detected by RT‐qPCR. (D) Flow cytometry analysis of SPP1 expression in SAMs. (E) Representative immunofluorescence images of SPP1 in SAMs. Scale bar: 20 µm. (F) Gene expression of *Cx3cr1* detected by RT‐qPCR and Flow cytometry analysis of Cx3CR1 expression in SAMs. (G) Schematic diagram of the co‐culture of SAMs and primary lung fibroblasts. (H) Representative immunofluorescence images of FN1 in primary lung fibroblasts. Scale bar: 20 µm. [(A–D), (F)] Data show means ± SEM and are representative of at least three independent experiments. Symbols on bar graphs represent individual mice or independent experiments. [(A–D), (F)] one‐way ANOVA with Dunnett's multiple comparisons test was used. ^*^ *p* < 0.05, ^**^ *p* < 0.01, ^***^ *p* < 0.001 compared with the indicated groups.

Based on the characteristic gene signature of SAMs identified from sciRNA‐seq, we further validated the regulatory role of RIPK3 in modulating the key SAM markers *Spp1* and *Arg1* within in vivo models. Immunofluorescence staining revealed a marked increase in SPP1^+^ macrophages in fibrotic lung tissue compared to healthy controls (Figure S6A), with spatial colocalization in regions of alveolar collapse. In the BLM‐induced lung injury and fibrosis model, both *Spp1* and *Arg1* expression in lung tissue were significantly reduced in *Ripk3*‐CKO mice compared to *Ripk3*‐C controls (Figure 5A,B). Previous studies have established that SAM differentiation is closely linked to Granulocyte‐Macrophage Colony‐Stimulating Factor (GM‐CSF) [47, 50]. The underlying mechanism was thought to involve the physical proximity between GM‐CSF–producing neutrophils and macrophages in the scar; neutrophil‐derived GM‐CSF promotes the differentiation of monocytes into SAMs, while the key pro‐fibrotic cytokine Transforming Growth Factor‐beta (TGF‐β) further drives SAM maturation toward a pro‐fibrotic phenotype [47]. Furthermore, other research indicates that GM‐CSF is a more potent inducer of SPP1 expression in human monocytes compared to M‐CSF [58]. To mimic this pro‐fibrotic microenvironment in vitro, we cultured mouse bone marrow–derived monocytes with GM‐CSF for 9 days, followed by secondary stimulation with TGF‐β (Figure S6B). Macrophages differentiated from *Ripk3*‐CKO bone marrow exhibited loss of RIPK3, whereas TGF‐β treatment upregulated RIPK3 in *Ripk3*‐C cells (Figure S6C,D). Under TGF‐β stimulation, *Spp1* and *Arg1* levels rose substantially in *Ripk3*‐C macrophages but were markedly attenuated in *Ripk3*‐CKO cells (Figure 5C). This regulatory effect was further confirmed by flow cytometry and immunofluorescence analysis of SPP1 expression (Figure 5D,E). Given reports that TGF‐β upregulates Cx3CR1 expression in microglia and monocytes [59, 60] and that Spp1 can drive monocyte chemotaxis [61, 62], we assessed Cx3CR1 levels following TGF‐β treatment. Cx3CR1 expression increased significantly in *Ripk3*‐C macrophages but was substantially reduced in *Ripk3‐*CKO cells, suggesting that RIPK3 may regulate SAM migration or localization via Cx3CR1 (Figure 5F).

We next sought to mechanistically dissect whether this regulation of SAM signature genes relied on the canonical necroptotic activity of RIPK3. Western blot analysis revealed no detectable phosphorylation of MLKL or RIPK3 following TGF‐β stimulation (Figure S7A). Moreover, pharmacological inhibition of RIPK1/3 kinase activity failed to recapitulate the suppressive effects of RIPK3 deficiency on the expression of *Spp1*, *Arg1*, and *Cx3cr1* (Figure S7B). These data indicate that RIPK3 governs the acquisition of the pro‐fibrotic SAM phenotype through a kinase‐independent, non‐necroptotic mechanism.

To investigate the impact of SAMs on fibroblast activation, we co‐cultured TGF‐β‐activated SAMs with primary pulmonary fibroblasts (Figure 5G). TGF‐β–activated SAMs from *Ripk3*‐C mice significantly induced upregulation of *Col1a1*, *Fn1*, and *Col4a1* in fibroblasts, whereas SAMs from *Ripk3*‐CKO mice elicited markedly lower expression of these genes (Figure S6F,G). Immunofluorescence staining additionally demonstrated that RIPK3 ablation significantly reduced FN1 deposition by fibroblasts (Figure 5H). Together, these findings indicate that RIPK3 modulates SAM's functional status to promote fibroblast activation and extracellular matrix deposition, and that targeting RIPK3 may represent an effective strategy to interrupt this pro‐fibrotic cascade.

### 2.6 RIPK3 Regulates Arginine Metabolism and Polyamine Biosynthesis in SAMs

**FIGURE 6:**
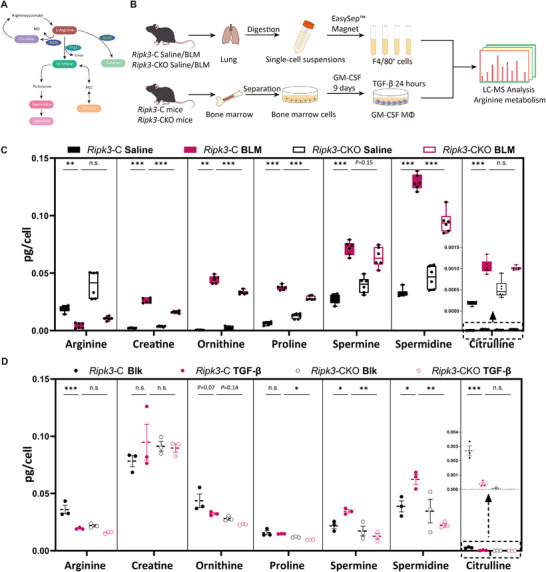
RIPK3 orchestrates polyamine‐biased arginine metabolism and PI3K‐AKT‐mTOR activation in SAMs. (A) Intracellular arginine metabolic pathway. (B) Flowchart illustrating the LC‐MS detection of arginine and related metabolite levels in pulmonary macrophages and SAMs. (C) LC‐MS detection of Arginine, Creatine, Ornithine, Proline, Spermine, Spermidine, and Citrulline levels in pulmonary macrophages. (D) LC‐MS detection of arginine and related metabolite levels in SAMs. (C,D) Data show means ± SEM and are representative of at least three independent experiments. Symbols on bar graphs represent individual mice or independent experiments. (C,D) One‐way ANOVA with Dunnett's multiple comparisons test was used. ^*^ *p* < 0.05, ^**^ *p* < 0.01, ^***^ *p* < 0.001 compared with the indicated groups. n.s., not significant.

Based on the metabolic function of the SAM‐characteristic gene *Arg1*, we further elucidated the regulatory role of RIPK3 in arginine metabolism. We first conducted a metabolic pathway analysis based on the sciRNA‐seq data. This revealed distinct metabolic states between control and *Ripk3*‐CKO SAMs. Notably, pathways related to “Arginine and proline metabolism” were downregulated in *Ripk3*‐CKO SAMs, suggesting that RIPK3 is a key transcriptional regulator of this axis (Figure S8). Based on this finding and the metabolic function of the SAM‐characteristic gene *Arg1*, we further elucidated the regulatory role of RIPK3 in arginine metabolism. The intracellular metabolic pathways of arginine are complex, primarily involving the urea cycle, nitric oxide synthesis, polyamine synthesis, ornithine and proline synthesis, and creatine synthesis (Figure 6A). To investigate the dynamic changes in arginine metabolism within macrophages during pulmonary fibrosis and the regulatory role of RIPK3, a multi‐level metabolic analysis strategy was employed. First, the arginine metabolic profile of F4/80^+^ macrophages from the lung tissues of mice with pulmonary fibrosis was examined using LC‐MS (Figure 6B). Compared to the saline group, the arginine content in F4/80^+^ macrophages from fibrotic lung tissue was significantly reduced, while its downstream metabolites (creatine, ornithine, proline, spermine, spermidine, and citrulline) all showed characteristic accumulation (Figure 6C). This phenomenon suggested that the arginine metabolism in macrophages within the pulmonary fibrotic microenvironment is activated through multiple pathways, potentially promoting fibrotic progression. In the lung tissue of *Ripk3*‐CKO BLM mice, arginine levels were partially restored, and its metabolites, including creatine, ornithine, proline, and polyamines, were significantly decreased, indicating that RIPK3 participates in the pro‐fibrotic process by regulating arginine metabolism (Figure 6C). As the aforementioned data reflected the metabolic state of the total macrophage population, and considering the cellular heterogeneity of metabolic changes, we further focused on SAMs. Utilizing an in vitro model of SAMs induced by GM‐CSF/TGF‐β combined with LC‐MS analysis (Figure 6B), it was found that TGF‐β stimulation led to a significant decrease in intracellular arginine levels in SAMs, with no significant impact on the creatine and proline metabolic pathways. Notably, TGF‐β specifically activated the polyamine synthesis pathway, inducing ornithine consumption and abnormal accumulation of spermine and spermidine, an effect that was significantly inhibited by RIPK3 knockout (Figure 6D). To determine the molecular basis for these metabolic shifts, we analyzed the expression of rate‐limiting enzymes in the polyamine biosynthetic pathway. Consistent with the metabolite profiles, qPCR analysis revealed that TGF‐β induced the expression of ornithine decarboxylase 1 (*Odc1*) and spermine synthase (*Sms)*, whereas RIPK3 deficiency significantly blunted their upregulation (Figure S8C). This suggests that RIPK3 controls polyamine accumulation by regulating the transcription of key biosynthetic enzymes.

Considering that TGF‐β drives SAM maturation toward a pro‐fibrotic phenotype, we examined how RIPK3 modulates its signaling cascade. The TGF‐β pathway can be divided into the canonical Smad‐dependent axis and noncanonical Smad‐independent routes. Our data demonstrate that RIPK3 deletion does not alter SMAD2/3 phosphorylation or nuclear translocation (Figure S9A,B), suggesting that RIPK3 regulates SAM function via a noncanonical mechanism. Previous studies have shown that the PI3K‐AKT‐mTOR pathway, a recognized noncanonical TGF‐β signaling branch, activates the nuclear transcription factor LXR to upregulate ARG1 transcription, and also plays a critical role in cellular metabolism [63, 64, 65, 66]. Given RIPK3's known influence on arginine metabolism, we hypothesized that RIPK3 may impinge on this pathway. Indeed, RIPK3 ablation markedly reduced TGF‐β–induced phosphorylation of AKT, p70S6K, and 4E‐BP1 (Figure S9C–E), indicating that RIPK3 is a pivotal upstream regulator of PI3K‐AKT‐mTOR activation. To definitively establish the signaling hierarchy, we performed rescue experiments. Exogenous polyamine supplementation successfully restored SAM marker expression in RIPK3‐deficient SAMs (Figure S9F), identifying polyamines as key downstream effectors. Furthermore, blocking the AKT pathway via PI3K inhibition in wild‐type SAMs recapitulated the *Ripk3*‐CKO phenotype, but caused no further suppression in RIPK3‐deficient SAMs (Figure S9G). Collectively, these data support a linear pathway where RIPK3 functions upstream of PI3K‐AKT to drive arginine‐polyamine metabolism.

### 2.7 RIPK3 Knockdown in Lung Tissue Ameliorates BLM‐Induced Pulmonary Fibrosis

**FIGURE 7:**
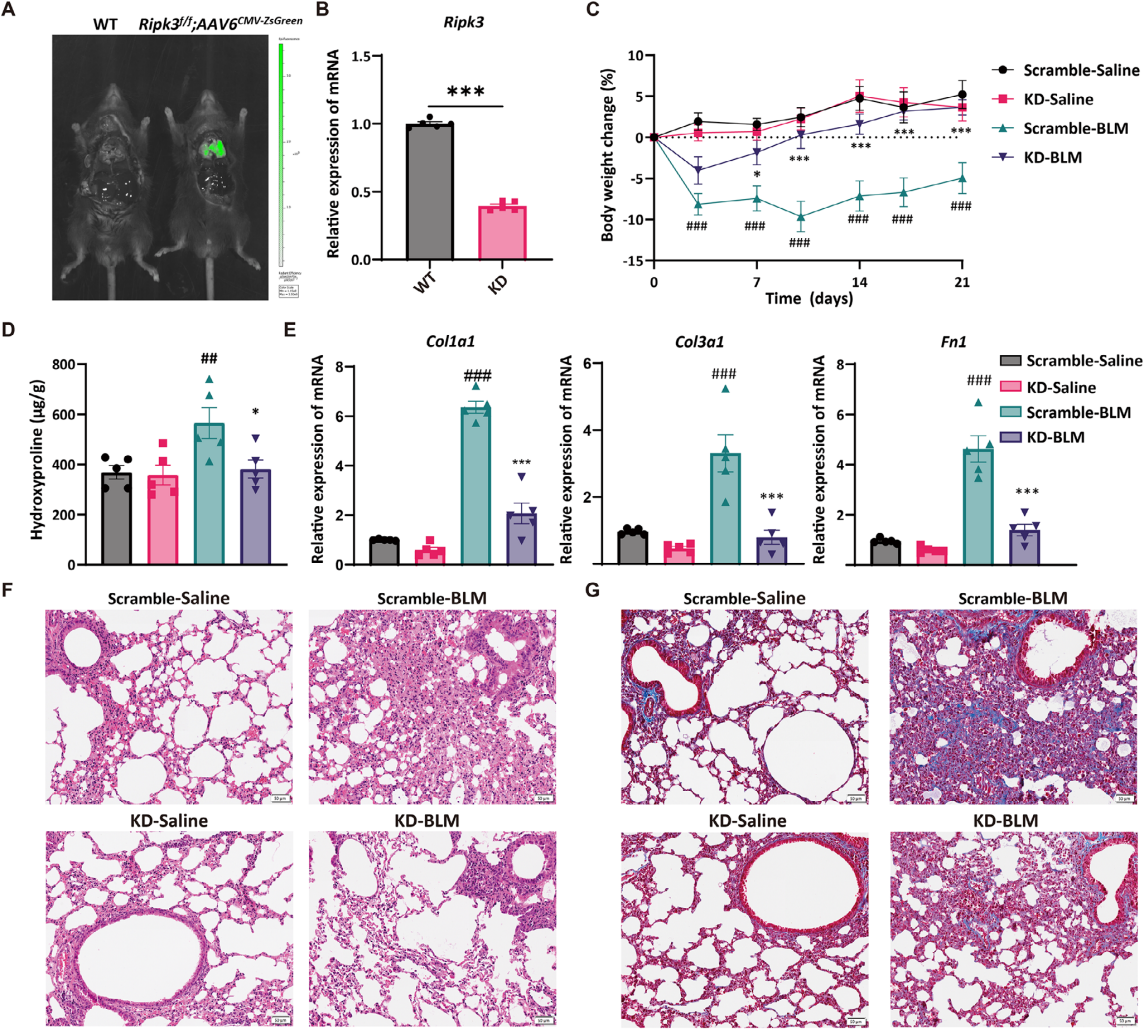
Lung‐specific RIPK3 knockdown ameliorated BLM‐induced pulmonary fibrosis. (A) In vivo fluorescence imaging detects ZsGreen expression intensity in mouse lungs. (B) PCR identification of gene recombination in lung tissue from Vector and *Ripk3*‐KD mice. (C) Body weight changes in mice with BLM‐induced pulmonary fibrosis. (D) Hydroxyproline content in lung tissue from mice with pulmonary fibrosis. (E) RT‐qPCR analysis of *Col1a1*, *Col3a1*, and *Fn1* expression levels in mouse lungs. (F,G) Representative images of H&E staining and Masson's trichrome staining of lung tissue (scale bar: 50 µm). (B–E) Data show means ± SEM. Symbols on bar graphs represent individual mice. (B) unpaired two‐tailed Student's *t*‐test, (C) two‐way ANOVA with Dunnett's multiple comparisons test, (D,E) one‐way ANOVA with Dunnett's multiple comparisons test were used. ^##^ *p* < 0.01, ^###^ *p* < 0.001, compared with the Scramble‐Saline group. ^*^ *p* < 0.05, ^***^ *p* < 0.001 compared with the Scramble‐BLM group.

Based on our findings regarding the role of RIPK3 in regulating SAM metabolic function, we further explored therapeutic strategies targeting RIPK3 for pulmonary fibrosis. To investigate pulmonary RIPK3 intervention as a therapeutic approach for IPF, we constructed an adenoviral vector expressing Cre recombinase under the CMV promoter. This vector was administered via intratracheal instillation into *Ripk3^flox/flox^* mice to achieve lung‐specific knockdown of RIPK3 (designated *Ripk3*‐KD). Fluorescence tracing using a control adenovirus expressing ZsGreen under the CMV promoter (CMV‐ZsGreen, designated scramble) was performed. Strong green fluorescence was observed predominantly in the lung tissue following intratracheal administration, with minimal signal detected in other major organs such as the heart, liver, and kidney, confirming efficient and lung‐targeted adenoviral delivery (Figure 7A). RT‐qPCR analysis of *Ripk3* confirmed the efficacy of the knockdown strategy (Figure 7B). We then subjected *Ripk3*‐KD mice to BLM‐induced fibrosis. *Ripk3*‐KD animals exhibited attenuated body‐weight loss throughout the injury (Figure 7C). Consistently, *Ripk3*‐KD mice exhibited ameliorated fibrosis, evidenced by reduced hydroxyproline levels and diminished collagen deposition area as confirmed by histological and quantitative analyses (Figure 7D,F,G; Figure S10B). This therapeutic efficacy was further corroborated by substantial decreases in canonical fibrotic gene expression (*Col1a1*, *Col3a1*, *Fn1*) and FN1 protein abundance (Figure 7E; Figure S10A). Collectively, these data indicated that pan‐pulmonary knockdown of RIPK3 effectively suppresses BLM‐induced fibrogenesis, providing both experimental proof‐of‐concept and theoretical support for targeting RIPK3 in IPF.

## 3 Discussion

**FIGURE 8:**
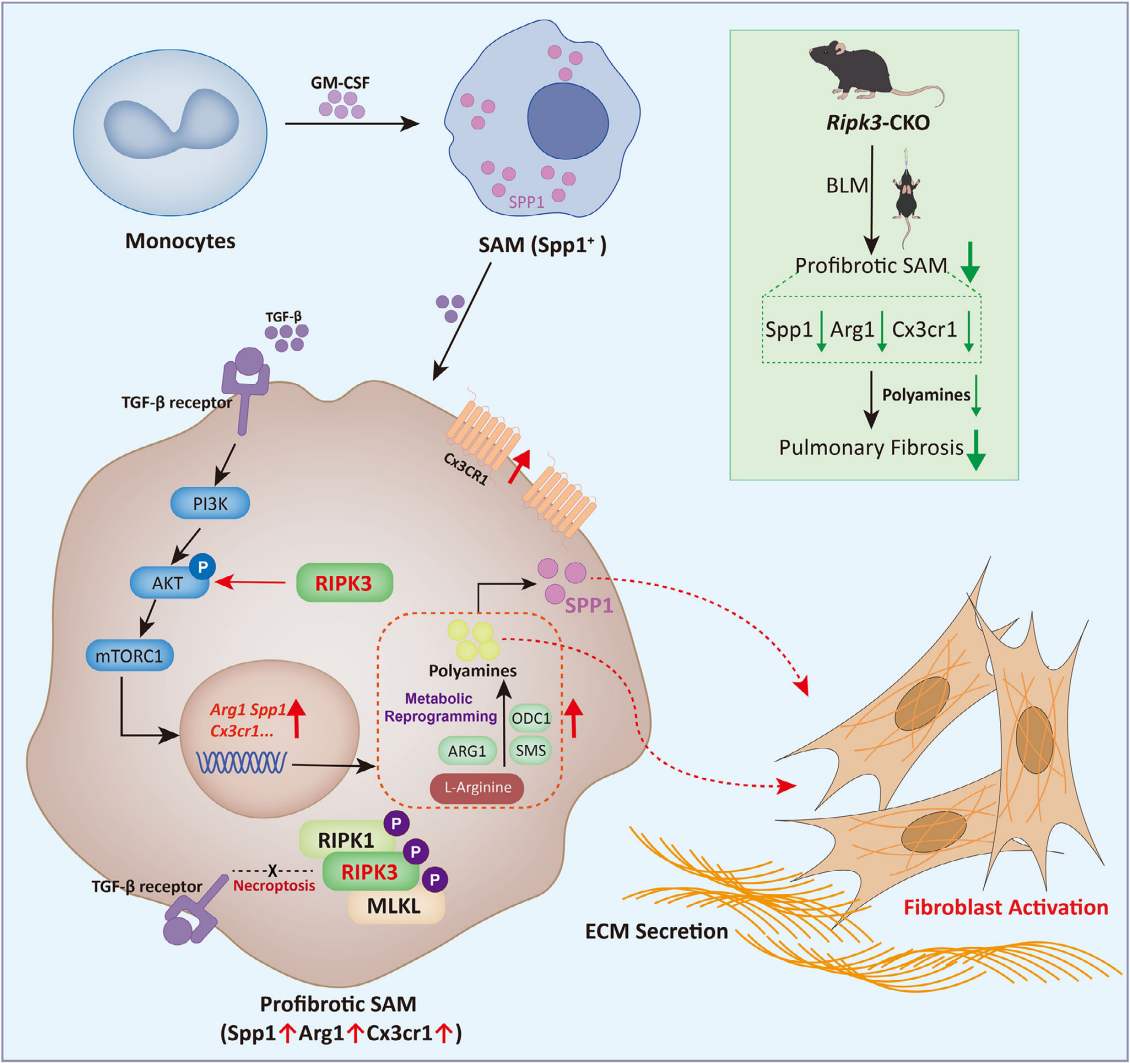
Schematic model of the RIPK3‐mediated immunometabolic axis in pulmonary fibrosis. Upon TGF‐β stimulation, RIPK3 is upregulated in SAMs. Distinct from its canonical role in necroptosis, RIPK3 functions in a kinase‐independent manner to recruit and activate the PI3K‐AKT‐mTOR signaling cascade. This signaling axis drives the sequential upregulation of key metabolic enzymes, including Arginase 1 and polyamine biosynthetic enzymes (ODC1, SMS). Consequently, ARG1 facilitates the rapid conversion of arginine into ornithine, which is subsequently metabolized by ODC1 and SMS into polyamines. The resulting intracellular polyamine accumulation sustains the high expression and secretion of SPP1. Secreted SPP1 and polyamine act in a paracrine manner to activate fibroblasts and promote myofibroblast differentiation.

RIPK3, a pivotal molecule in the necroptosis signaling pathway, has long been functionally oversimplified as merely a relay component within the RIPK1‐MLKL axis. Consequently, therapeutic interventions targeting necroptosis have predominantly focused on upstream RIPK1 inhibitors or downstream MLKL blockers, leading to an underestimation of RIPK3's diverse biological functions and pathological significance. However, emerging research in recent years has progressively unveiled its pleiotropic roles: beyond necroptosis, RIPK3 could participate in inflammasome activation, autophagy regulation, and cellular metabolism through necroptosis‐independent mechanisms. Unlike RIPK1 knockout, which results in embryonic lethality, RIPK3‐deficient mice develop normally, offering a unique advantage for in vivo investigation of its non‐death‐dependent functions. Building upon this, the present study focuses on the immunometabolic regulatory mechanisms of RIPK3 in pulmonary fibrosis, aiming to provide a theoretical basis for developing precise RIPK3‐targeted therapeutic strategies. Based on our findings, we propose a novel model of immunometabolic crosstalk where RIPK3 acts as a molecular switch governing the pro‐fibrotic function of SAMs. Mechanistically, our data place RIPK3 upstream of the PI3K‐AKT signaling cascade. Upon TGF‐β stimulation, upregulated RIPK3 triggers AKT phosphorylation, which orchestrates the critical rewiring of arginine metabolism toward polyamine synthesis. These intracellular polyamines then serve as essential effectors to sustain the high expression and secretion of SPP1. Consequently, we postulate that SAMs drive fibrosis via a dual‐hit mechanism involving both the provision of instructive signals through SPP1‐mediated paracrine activation and the potential supply of secreted polyamines as metabolic substrates to support the high bioenergetic and biosynthetic demands of myofibroblast proliferation. This axis creates a feed‐forward loop that perpetuates the fibrotic response, as summarized in the schematic diagram presented in Figure 8.

Pulmonary fibrosis, a representative and often fatal interstitial lung disease, is pathologically characterized by aberrant fibroblast activation and excessive extracellular matrix deposition. Although currently approved drugs such as nintedanib and pirfenidone could slow disease progression, they offer limited improvement in overall survival, highlighting the failure of current therapeutic strategies to effectively target the core pathogenic drivers of the disease. The pathogenesis of IPF involves an imbalance between inflammatory injury and the subsequent repair processes, with patient outcomes contingent upon the bidirectional regulation of inflammation and fibrosis. Notably, macrophages, as the most abundant immune cell population in the lungs, play a dual role in the fibrotic process: initially exacerbating tissue damage through pro‐inflammatory responses, and later driving pathological repair by secreting pro‐fibrotic factors. Therefore, elucidating the dynamic roles of immune cells, particularly macrophages, within the fibrotic microenvironment is a critical avenue for overcoming existing therapeutic bottlenecks.

The functional heterogeneity of macrophages, the most abundant immune cell population in the lung, holds a central position in the progression of fibrosis [67]. While the traditional M1/M2 polarization model is widely cited, its explanatory power for complex pathological scenarios is limited. In this study, utilizing single‐cell RNA sequencing, we revealed a specific expansion of SAMs in pulmonary fibrosis. Their characteristic gene signature (including S*pp1*, *Gpnmb*, *Trem2*, *Cd9*, *Fabp5*, *Cd63*, and *Arg1*) is highly consistent with previously reported pro‐fibrotic macrophage phenotypes [47, 48, 49, 50, 51, 52, 53, 55]. Recent publications on spatial transcriptomics in pulmonary fibrosis have further elucidated the transcriptomic features of distinct regions within specific fibrotic niches, with multiple reports highlighting the accumulation of SPP1^+^ macrophages in fibrotic areas; indeed, increased *Spp1* expression correlates with pathological severity [67, 68, 69]. Beyond pulmonary fibrosis, SAMs have been observed in various diseases, such as pneumonia, liver fibrosis, hepatocellular carcinoma, cardiovascular diseases, and kidney injury. However, the precise mechanisms underlying their pathological roles remain controversial, primarily due to the scarcity of specific genetic tools and limitations of in vitro models. Our study successfully recapitulated the in vivo phenotype of SAMs by co‐inducing murine bone marrow‐derived monocytes with GM‐CSF and TGF‐β, providing a reliable model for dissecting SAM functions. Previous research has largely suggested that alveolar macrophages depend on GM‐CSF and TGF‐β for their development and homeostasis, while peripheral monocytes differentiate in response to M‐CSF before migrating to the alveoli to replace existing macrophages [44, 46, 59, 70]. However, peripheral monocytes may also be influenced by GM‐CSF and TGF‐β upon entering the pulmonary interstitium, leading to their differentiation into cells with SAM‐like phenotypes, thereby providing a rationale for our use of GM‐CSF to induce bone marrow‐derived cells [58].

In recent years, metabolic reprogramming has increasingly been recognized as a key mechanism governing macrophage phenotype switching and functional execution. Particularly in the context of fibrotic pathology, metabolic reprogramming may serve as a critical bridge linking immune regulation and fibrotic progression [71, 72]. For instance, M1 macrophages rely on glycolysis and the pentose phosphate pathway to meet their energy demands and support their pro‐inflammatory functions, whereas M2 macrophages predominantly utilize oxidative phosphorylation and fatty acid oxidation for energy production [73, 74, 75]. Furthermore, alterations in amino acid metabolism, especially arginine metabolism, in macrophages play a crucial role in fibrotic diseases [63, 64, 65, 66, 76, 77, 78]. Arginine serves as a precursor for proline and polyamine synthesis; proline is an essential component of collagen, and polyamines indirectly promote collagen synthesis by enhancing amino acid metabolism, regulating the activity of key enzymes, and activating the TGF‐β signaling pathway [79, 80]. Two main isoforms of arginase, ARG1 and ARG2, are involved in proline and polyamine generation. Studies have indicated that both *Arg1* and *Arg2* expression are induced during BLM‐induced pulmonary fibrosis [64, 78, 81]. By integrating single‐cell transcriptomic sequencing with metabolomic analysis, our study reveals for the first time that RIPK3 promotes arginine metabolism and a pro‐fibrotic phenotype in SAMs by modulating the TGF‐β‐Arg1 signaling axis. In pulmonary macrophages from *Ripk3*‐CKO mice with fibrosis, levels of arginine metabolites, including creatine, ornithine, proline, and polyamines (spermine, spermidine), were significantly reduced. In vitro experiments further confirmed that TGF‐β stimulation specifically activates the polyamine synthesis pathway in SAMs, leading to an abnormal accumulation of spermine and spermidine, a process abrogated by RIPK3 deletion. Concurrently, our preliminary investigation into *Arg2* expression in SAMs showed low basal levels and no induction upon TGF‐β stimulation (data not shown), further underscoring the importance of Arg1 in regulating SAM arginine metabolism.

Furthermore, employing an in vitro model of SAMs induced by GM‐CSF/TGF‐β, this study posits that RIPK3 could regulate the metabolic phenotype of SAMs through a death‐independent pathway: activation of the PI3K‐AKT‐mTOR pathway promotes *Arg1* expression, thereby driving the conversion of arginine to polyamines. This mechanism contrasts with the previously established pro‐necroptotic function of RIPK3, highlighting the cell‐type dependency of its functional pathways. Collectively, our mechanism‐based rescue experiments firmly established a linear signaling axis where TGF‐β‐induced *Ripk3* activation acts upstream of the PI3K‐AKT pathway to drive arginine‐polyamine metabolism. Specifically, we demonstrated that metabolic rescue with exogenous polyamines effectively restored the pro‐fibrotic phenotype in RIPK3‐deficient SAMs, whereas pharmacological blockade of PI3K in WT cells recapitulated the defects observed in RIPK3‐deficient cells. Conventional genetic rescue approaches, such as the overexpression of constitutively active AKT, will help to corroborate a more refined mechanism, the inherent resistance of primary macrophages to lipid‐based transfection and the potential for unintended alteration of their polarization states presented significant technical hurdles in the current study. Future investigations utilizing macrophage‐specific viral delivery systems or knock‐in models will be instrumental in further dissecting the precise kinase‐substrate interactions governing this non‐canonical RIPK3 function.

To validate the clinical translational potential of our findings, we employed an adenoviral vector to achieve local RIPK3 knockdown in the lungs. RIPK3 is highly abundant in pulmonary parenchymal cells, and multiple studies have demonstrated its involvement in necroptosis in lung epithelial cells, endothelial cells, and macrophages, thereby exacerbating disease progression. However, our research primarily focuses on the non‐necroptotic function of RIPK3. Accordingly, in establishing the adenoviral knockdown model, we carefully considered RIPK3's dual role in cell death and non‐death regulation. We utilized an adenoviral vector engineered for efficient lung targeting, administered via intratracheal instillation for organ‐restricted intervention. The ubiquitous CMV promoter was used to ensure effective silencing of RIPK3 expression within all pulmonary cells. The results demonstrated that lung‐specific knockdown of RIPK3 significantly ameliorated the fibrotic phenotype. This outcome not only supports the potential of local RIPK3 targeting as a novel therapeutic strategy for IPF but also provides a partial theoretical basis for future studies on how to finely modulate RIPK3 signaling pathways and differentiate its cell death‐related versus non‐death‐related effects.

It is instructive to juxtapose our current findings with the emerging metabolic roles of RIPK3 in other fibrotic organs, which reveals a remarkable degree of tissue specificity. In the context of liver fibrosis, Afonso et al. recently demonstrated that RIPK3 exacerbates non‐alcoholic steatohepatitis not only through necroptosis but also by rewiring lipid metabolism via PPARγ suppression [25]. Similarly, in kidney fibrosis, RIPK3 has been shown to act as a scaffold to activate the AKT‐ATP citrate lyase (ACL) signaling axis in fibroblasts, thereby promoting fatty acid synthesis and fibrogenesis [82]. In striking contrast to these lipid‐centric mechanisms, our study uncovers a unique scenario in pulmonary fibrosis where the RIPK3‐AKT axis is specifically activated within the immune compartment (macrophages) and is rewired to drive arginine‐polyamine metabolism. This dichotomy suggests that RIPK3 functions as a versatile metabolic hub that orchestrates fibrosis through distinct metabolic checkpoints—ranging from lipid synthesis in the liver and kidney to polyamine generation in the lung—depending on the specific microenvironmental cues.

Although the *Cx3cr1*‐Cre driver is widely employed to target mononuclear phagocytes, Cx3CR1 expression is not exclusively restricted to the macrophage lineage and can also occur in specific subsets of dendritic cells (DCs) and NK cells. However, several lines of evidence support the conclusion that the anti‐fibrotic phenotype observed in our RIPK3‐deficient mice is primarily driven by macrophage dysfunction. First, our isolation of F4/80^+^ lung macrophages revealed a robust and specific upregulation of RIPK3 in the fibrotic group, confirming that macrophages are a major cellular compartment exhibiting aberrant RIPK3 expression in this pathological context. Second, previous fate‐mapping studies in the bleomycin model have established that the Cx3CR1‐positive population in the fibrotic lung is overwhelmingly dominated by recruited monocyte‐derived macrophages, whereas neutrophils are largely Cx3CR1‐negative [83, 84]. Therefore, while we cannot completely rule out minor contributions from other Cx3CR1‐expressing lineages, the profound phenotypic changes and the specific molecular validation in sorted macrophages strongly implicate the macrophage compartment as the key effector of RIPK3‐mediated fibrosis.

Despite the valuable insights provided by this study, several aspects warrant further investigation to enhance the depth and translational potential of the findings. First, while the *Cx3cr1*‐Cre driver mouse is commonly used, it may also affect monocyte precursors, highlighting the need for more specific Cre lines to accurately trace the ontogeny of SAMs. Second, the arginine metabolic network is highly complex, involving multiple interconnected pathways; thus, the application of isotope tracing would be beneficial to more precisely define metabolic flux. Third, the specific mechanism by which the PI3K‐AKT‐mTOR pathway selectively regulates Arg1, rather than other metabolic genes, remains to be clarified. Additionally, whether RIPK3 modulates this pathway through its kinase activity, scaffold function, or by engaging non‐canonical TGF‐β signaling components, remains to be elucidated. Fourth, considering the limited applicability of adenoviral‐Cre systems in clinical settings, the development of adenoviral vectors carrying siRNAs may offer a more feasible approach for therapeutic validation. Fifth, this study relied primarily on genetic manipulation to probe RIPK3 function; integrating small‐molecule inhibitors, PROTACs, or combinatorial metabolic interventions in future studies may uncover potential synergistic effects. Lastly, as this work focused on RIPK3's role in arginine metabolism, comprehensive metabolomic profiling could reveal broader metabolic reprogramming events, including possible alterations in glycolysis and fatty acid oxidation.

In summary, this study elucidates that RIPK3 influences the PI3K‐AKT‐mTOR pathway to regulate arginine metabolism and the pro‐fibrotic functions of SAMs, thereby revealing its pivotal role in pulmonary fibrosis. Furthermore, the significant therapeutic efficacy of lung‐specific RIPK3 knockdown provides experimental support for developing novel therapeutic strategies for IPF. These findings not only expand the known biological functions of RIPK3 but also offer a new target for developing fibrosis therapies based on immunometabolic regulation. Future research could explore the generalizability of this pathway in other fibrotic diseases, such as liver and cardiac fibrosis, and combine multi‐omics technologies with clinical translation tools to pave new avenues for the precision treatment of chronic fibrotic diseases.

## 4 Experimental Section

### 4.1 Animals

Male C57BL/6J mice (8 weeks old, weighing 23–25 g) used in this experiment were purchased from Huachuang Sino Pharmaceutical Technology Co., Ltd. *Cx3cr1*‐Cre, *Ripk3^flox/folx^*, and *Rosa26^stop(flox/flox)Ripk3^* mice were kindly provided by Professor Haiyan Zhang's laboratory at the Shanghai Institute of Materia Medica, Chinese Academy of Sciences. Both experimental and breeding animals were housed in the specific‐pathogen‐free (SPF) animal facility at the Shanghai Institute of Materia Medica, Chinese Academy of Sciences. All experimental procedures were performed following the Guide for the Care and Use of Laboratory Animals and were approved by the Animal Ethics Committee of the Shanghai Institute of Materia Medica, Chinese Academy of Sciences. Experimental animals were acclimatized to the SPF barrier facility for at least one week before experimentation.

### 4.2 BLM‐Induced Lung Injury and Fibrosis

Mice were subjected to bleomycin (BLM) administration to induce pulmonary injury and fibrosis. Initially, a sterile saline solution was prepared to dissolve BLM to achieve a final concentration of either 0.85 or 2 mg/kg. The specific concentration was determined based on the experimental design and the body weight of mice. Subsequently, experimental mice were anesthetized using Zoletil (tiletamine‐zolazepam). Following confirmation of adequate anesthesia depth, mice were restrained. Bronchotracheal instillation was then performed using a pipette, with an instillation volume of 50 µL per mouse. The respiratory status of the mice was carefully monitored, and upon confirmation of no adverse reactions, the mice were returned to cages for recovery. Experimental endpoints were defined at days 10 and 21 post‐induction, and animal survival and body weight were continuously monitored.

### 4.3 GEO Database Analysis

Gene expression profiles were analyzed using data retrieved from the Gene Expression Omnibus (GEO) database. The GEO official website was accessed to search for RNA‐Seq datasets using GSE series accession numbers. Differential gene expression analysis was subsequently performed on the selected datasets using the online analytical tool GEO2R.

### 4.4 Isolation of M‐CSF‐Induced Bone Marrow‐Derived Macrophages (BMDMs)

Mice were euthanized, and the mouse surface was subsequently sterilized. Femurs and tibias were dissected from the animals and transferred to serum‐free Iscove's Modified Dulbecco's Medium (IMDM). The ends of the bones were cut, and the bone marrow was flushed out using a syringe; cells were then dispersed uniformly by gentle pipetting. The suspension was filtered through a 100 µm cell strainer, and cells were pelleted (1200 rpm, 5 min). After supernatant removal, Red Blood Cell (RBC) Lysis Buffer was added and gently agitated for 4 min. Lysis was terminated with IMDM, and cells were re‐pelleted; the supernatant was discarded. The pellet was resuspended in IMDM with 10% Fetal Bovine Serum (FBS), filtered again through a 100 µm cell strainer, and adjusted to 2 × 10^6^ cells/mL in IMDM with 10% FBS and 10 ng/mL M‐CSF. Cells were seeded into culture plates or dishes. On day 3 post‐seeding, non‐adherent cells were removed, and fresh M‐CSF‐containing medium was added. By day 7, differentiated macrophages were ready for use.

### 4.5 Isolation of GM‐CSF‐Induced Bone Marrow‐Derived Macrophages

GM‐CSF‐induced bone marrow‐derived macrophages (GM‐CSF‐BMDMs, SAMs) were generated by culturing bone marrow cells with granulocyte‐macrophage colony‐stimulating factor (GM‐CSF). Femurs and tibias of mice were dissected and transferred to serum‐free RPMI 1640 medium. The bone marrow contents were flushed out using a syringe and gently dispersed by pipetting. After supernatant removal, Red Blood Cell Lysis Buffer was added and gently agitated for 4 min. The cell suspension was adjusted to a concentration of approximately 2 × 10^6^ cells/mL in RPMI 1640 medium with 10% FBS supplemented with 20 ng/mL recombinant murine GM‐CSF. Cells were seeded into pre‐prepared cell culture plates or dishes for subsequent culture. On days 3 and 6 of culture, the GM‐CSF‐containing medium was replaced to remove non‐adherent, non‐target cells. By day 9, monocytes had fully differentiated into macrophages, and these GM‐CSF‐BMDMs were then ready for downstream experimental procedures.

### 4.6 Peritoneal Macrophage Isolation

Primary peritoneal macrophages were isolated from mice using peritoneal lavage. Under sterile conditions, 6–8 mL/20 g body weight of pre‐chilled sterile RPMI 1640 medium was slowly injected into the peritoneal cavity using a 10 mL syringe. After injection, the abdomen was gently massaged for 1–2 min to facilitate cell suspension within the peritoneal cavity. Subsequently, mice were tilted, and the peritoneal lavage fluid was slowly aspirated using the same syringe, taking care to avoid puncturing the intestines or blood vessels. The collected lavage fluid was transferred to a 15 mL centrifuge tube and centrifuged at 300 × g for 5 min at 4°C. After discarding the supernatant, the cell pellet was gently resuspended in complete RPMI 1640 medium supplemented with 10% FBS. The cell suspension was evenly seeded into 6‐well culture plates or cell culture flasks and incubated at 37°C for 1 h to purify macrophages by utilizing their adherence properties. Following adherence, the culture medium was removed to eliminate non‐adherent cells. The adherent macrophages were gently washed twice with pre‐warmed phosphate‐buffered saline (PBS) and cultured in fresh complete medium.

### 4.7 Alveolar Macrophage Isolation

Alveolar macrophages were obtained using bronchoalveolar lavage fluid (BALF), and the cell pellet was resuspended in complete RPMI 1640 medium supplemented with 10% FBS. The cell suspension was evenly seeded into 6‐well culture plates or cell culture flasks and purified by utilizing the adherence properties of macrophages.

### 4.8 Lung Tissue Digestion and Flow Cytometry Analysis

Digestion buffer was prepared using RPMI 1640 medium supplemented with 10% FBS, containing collagenase type IV (1.5 mg/mL) and DNase I (0.04 mg/mL). Mouse lung tissues were harvested and placed in dishes. The prepared digestion buffer was then added, and the lung tissues were minced into a paste‐like consistency using scissors to facilitate enzymatic digestion. The dishes were placed in a 37°C incubator and incubated for 1 h, with gentle agitation of the dishes periodically to promote digestion. The lung tissues were homogenized using glass slides and filtered through a 100 µm cell strainer to remove undigested tissue fragments. Red blood cell lysis buffer was added to the cell suspension. The cell suspension was further filtered through a 70 µm cell strainer for a second filtration to further remove debris. For flow cytometry staining, the prepared single‐cell suspension was adjusted to a concentration of 2 × 10^6^ cells per tube and transferred into flow cytometry tubes. FACS washing buffer was added, and the tubes were centrifuged at 400 × g for 6 min. After centrifugation, the samples were washed once with PBS. Fixable Viability Dye (FVD) was added to the samples, and the samples were incubated at 4°C in the dark for 30 min for live/dead cell discrimination in subsequent experiments. Subsequently, FACS washing buffer was added to each sample. Anti‐CD16/32 antibody was added to block non‐specific binding epitopes of the antibody Fc region. After another centrifugation and removal of the washing buffer, fluorescence‐labeled antibodies were added, and the samples were incubated in the dark at 4°C for 20 min. Following incubation, cells were washed with FACS washing buffer and centrifuged as described above. After centrifugation, the cells were resuspended in an appropriate volume of FACS washing buffer and were ready for flow cytometric analysis. Flow cytometric analysis was performed using a BD LSR Fortessa flow cytometer, and FlowJo 10 software was used for data analysis.

### 4.9 RNA Isolation and Real‐Time Quantitative PCR

Total RNA was extracted from tissues and cells using the Total RNA Extraction Purification Kit from Tiangen Biotech (Beijing, China) and strictly following the manufacturer's instructions. Subsequently, cDNA was synthesized from RNA samples using the Hifair AdvanceFast One‐step RT‐gDNA Digestion SuperMix for qPCR from Yeasen Biotech Co., Ltd. (Shanghai, China). Real‐time quantitative PCR (RT‐qPCR) analysis was performed using the SYBR Green Realtime PCR Master Mix from Yeasen and the 7500 Fast Real‐Time PCR System (Applied Biosystems, Foster City, CA, USA) with gene‐specific primers. The sequences of primers for RT‐qPCR are listed in Table S1.

### 4.10 Western Blot Analysis

Tissue samples (approximately 10 mg) were initially homogenized in sodium dodecyl sulfate (SDS) lysis buffer containing protease and phosphatase inhibitors. For cell samples, cells were lysed by repeated pipetting in SDS lysis buffer. Protein denaturation was performed using a metal bath at 100°C for 10 min. Equal amounts of protein from each sample were then separated by SDS‐PAGE. Proteins were subsequently transferred to polyvinylidene difluoride (PVDF) membranes using a Trans‐Blot Turbo Transfer System. Membranes were blocked with 5% skim milk/TBST (1 h), incubated with primary antibodies (overnight, 4°C), washed, and incubated with HRP‐secondary antibodies (1 h, RT). After washing, the chemiluminescent substrate was added, and target protein expression was then detected using a Bio‐Rad gel imaging system.

### 4.11 Lung Histopathological Examination

Mouse lung tissues underwent fixation in 4% paraformaldehyde, followed by paraffin embedding and sectioning. For histopathological examination, the paraffin sections were stained with hematoxylin and eosin (H&E) and Masson's trichrome.

### 4.12 Immunofluorescent Staining

Cells were seeded on slides in 12‐well plates for overnight adherence. Cells were washed with PBS, fixed (10 min, RT), washed, and blocked with immunofluorescence blocking solution (1 h, RT). Primary antibody was added (overnight, 4°C). After washing, fluorescence‐labeled secondary antibody was added (1 h, RT). Cells were washed, nuclei counterstained with DAPI, and mounted with anti‐fade medium for imaging using a Leica TCS SPS CFSMP fluorescence microscope. Paraffin sections were dewaxed in xylene (3 times), rehydrated in graded ethanol solutions, and rinsed in water. Antigen retrieval was performed in preheated antigen retrieval solution (20 min, 95°C–100°C), followed by cooling and washing. Hydrophobic barriers were drawn, and immunostaining (blocking, antibody incubations) was performed similarly to cell immunofluorescence.

### 4.13 Hydroxyproline Detection

Hydroxyproline (HYP) content in lung tissue was measured using a Hydroxyproline Assay Kit (Nanjing Jiancheng). Absorbance was measured at 550 nm using a microplate reader. Hydroxyproline concentrations in samples were calculated based on the standard curve.

### 4.14 Assessment of Murine Pulmonary Function

The Whole Body Plethysmography (WBP) system is a cutting‐edge, non‐invasive technology widely used to assess pulmonary function in small experimental animals, particularly awake mice. Prior to experimentation, the WBP system requires systematic calibration and background noise acquisition. Before formal testing, experimental mice undergo acclimation training within the plethysmography chamber. During the actual measurement, mice are gently placed into the plethysmography chamber, and data acquisition commences after their respiration stabilizes, recording respiratory data for 20 min.

### 4.15 Micro‐Computed Tomography

Mice were anesthetized with continuous monitoring of the depth of anesthesia and stability of vital signs. Mice were then precisely positioned and secured onto the Micro‐CT scanning bed. Scanning parameters, including voltage, current, and scan range, were configured, followed by initiating the scan program to acquire lung data. After data acquisition, dedicated reconstruction software was employed to convert raw projection data into 3D images.

### 4.16 Sorting of Lung Macrophages

First, prepare a single‐cell suspension of lung tissue following the method described above. Use the EasySep Mouse F4/80^+^ Cell Isolation Kit to isolate F4/80^+^ macrophages from the lung tissue single‐cell suspension via magnetic bead‐based positive selection. The processed cell suspension is filtered through a 40 µm cell strainer to obtain the final F4/80^+^ macrophage suspension.

### 4.17 sci‐RNA‐seq3 Library Preparation and Sequencing

RNA libraries were prepared using sci‐RNA‐seq3, optimized based on nuclear isolation [85]. F4/80^+^ sorted lung macrophages were lysed with freshly prepared hypotonic lysis buffer containing diethyl pyrocarbonate (DEPC) to inactivate RNases. Fixation was performed on ice for 15 min, followed by gradual rehydration in 0.3 m SPBSTM buffer. Nuclei from each mouse were distributed into a 96‐well plate (approximately 20 000 nuclei per well). Each well contained 2 µL 10 µm unique indexed oligo (dT) primer (5′‐/5Phos/CAGAGCNNNNNNNN[10bp‐barcode] TTTTTTTTTTTTTTTTTTTTTTTTTTTTTT‐3′, where “N” is any base) and 2 µL 10 mm dNTP mix (Thermo Fisher). After heat denaturation and primer annealing (55°C, 5 min), reverse transcription was performed using SuperScript IV reverse transcriptase. The RT mix included 0.5 µL SuperScript IV, 2 µL 5× SuperScript IV RT buffer, and 0.5 µL nuclease‐free water per well, and the reaction was incubated at 55°C for 10 min. We then collected nuclei, washed, and redistributed them to a second 96‐well plate for ligation. The indexed ligation primers (5’‐GCTCTG[9‐ or 10‐bp ligation index] TACGACGCTCTTCCGATCT[reverse complement of ligation index]‐3’) were ligated to the RT primers using 0.5 µL T4 DNA ligase and 1.5 µL 10× T4 ligase buffer for 20 min at room temperature. Then the nuclei were collected, washed with 0.3 m SPBSTM, and redistributed to a third 96‐well plate for second‐strand synthesis. For second‐strand synthesis (SSS), a master mix was prepared by combining 67.5 µL nuclease‐free water, 7.5 µL 10 × second‐strand buffer, and 25 µL 20× second‐strand enzyme mix. 1 µL of SSS mix was added to each well, and the reaction was incubated at 16°C for 2.5 h. Subsequently, 1 µL of protease was added to digest nuclear proteins and release cDNA, followed by incubation at 37°C for 30 min and heat inactivation at 75°C for 20 min. For tagmentation, each well was mixed with 4.7 µL 2 × TD buffer and 0.3 µL N7‐Tn5, and then incubated at 55°C for 5 min to carry out tagmentation. The transposases were then inactivated by adding 0.4 µL 1% (wt./vol.) SDS, 0.4 µL 2% BSA, and 1.8 µL nuclease‐free water. The mixture was incubated for 15 min at 55°C in a thermocycler, followed by quenching of SDS with the addition of 2 µL of 10% (vol/vol) Tween 20 to each well. For PCR amplification, each well was mixed with 2.5 µL of 10 µm indexed P5 primer (5’ ‐AATGATACGGCGACCACCGAGATCTACAC[PCR P5 index] ACACTCTTTCCCTACACGACGCTCTTCCGATCT‐3’), 2.5 µL of 10 µm indexed P7 primer (5’ ‐CAAGCAGAAGACGGCATACGAGAT[PCR P7 index]GTCTCGTGGGCTCGG‐3’), and 25 µL NEBNext High‐Fidelity 2 × PCR Master Mix. Nuclease‐free water was added to bring the final volume to 50 µL. The cDNA libraries were purified using 0.8 volumes of MagMAX Pure Bind, and 300–700 bp fragments were size‐selected by agarose gel extraction. Final libraries were sequenced on an Illumina NovaSeq X Plus system using paired‐end 150 bp reads. Base calling was performed using Illumina's standard pipeline and converted to FASTQ files. Demultiplexing, barcode and UMI extraction, read filtering, and alignment to the reference genome were conducted using a custom pipeline. Aligned reads were deduplicated based on UMI to generate the digital gene expression matrix.

### 4.18 Single‐Cell Seq Analysis

Raw sequencing data were first assessed using FastQC to evaluate overall read quality. Low‐quality bases and adapter sequences were subsequently removed using fastp, generating high‐quality clean reads. Clean reads were then aligned to the reference genome using STAR, and a gene expression matrix was generated. Cells were filtered using Seurat (v5.3.0) based on the number of detected genes and the percentage of mitochondrial gene expression. After quality control, a total of 8405 high‐quality cells were retained for further analysis. The expression matrix of filtered cells was normalized, and highly variable genes were identified using FindVariableFeatures implemented in Seurat. Principal component analysis (RunPCA) was applied for dimensionality reduction, followed by unsupervised clustering using FindClusters. Cluster‐specific marker genes were identified using FindAllMarkers, and cell types were annotated based on canonical marker genes. For trajectory inference, Slingshot was used to reconstruct differentiation lineages, revealing two main trajectories originating from Ly6C^−^ monocytes (Ly6C^−^Mo) and Ly6C^+^ monocytes (Ly6C^+^Mo) toward alveolar macrophages (AM). To model gene expression dynamics along pseudotime, tradeSeq was applied to identify genes with significant temporal expression changes across the trajectories. Pathway enrichment analysis was performed using clusterProfiler, including KEGG and GO enrichment analyses, to identify enriched biological pathways associated with upregulated or downregulated genes. Metabolic pathway activity was further evaluated using the scMetabolism. Standardized metabolic activity scores were calculated to compare RIPK3 knockout and control cells and used for downstream visualization.

### 4.19 Isolation of Murine Primary Lung Fibroblasts

Primary fibroblasts were isolated from neonatal mouse lungs by enzymatic digestion, following established literature methods [86].

### 4.20 Identification of Arginine Metabolites

Cell lysates were prepared by combining cell samples with 100 µL of ice‐cold 80% methanol/water solution. The cell lysis solutions were subjected to ultrasonic treatment for 15 min in ice‐cold water to ensure complete disruption of cellular membranes. For the analysis of Citrulline, Arginine, Ornithine, Spermidine, Spermine, and Proline, a portion of 15 µL from each standard mixture solution and cell lysis solution was transferred into the bottom of a tube. To this, 70 µL of sodium borate buffer (200 mm, pH 8.8) was added. Subsequently, 25 µL of 6‐aminoquinolyl‐N‐hydroxysuccinimidyl carbamate (AQC; 4 mg/mL in acetonitrile) was added to the tube, which was then sealed and heated at 55°C for 10 min to facilitate AQC–amino acid derivatization. After cooling to room temperature, the mixture was centrifuged for 5 min at 4000 rpm, and 0.5 µL of the supernatant was injected into the UPLC‐ESI‐MS system for analysis. For Creatine analysis, 10 µL of each standard solution and cell lysis solution were transferred into a 96‐well plate with 40 µL of water added to each well. The plate was shaken for 20 min and then centrifuged for 5 min at 4000 rpm. A volume of 1 µL from each sample was subsequently injected into the UPLC‐ESI‐MS system. The analyses were performed using a Waters ACQUITY UPLC H‐Class system (Waters, Milford, MA, USA) coupled to a triple quadrupole 6500 mass spectrometer (AB SCIEX, Framingham, MA, USA), equipped with an electrospray ionization (ESI) source. Chromatographic separation was achieved using an Acquity UPLC BEH T3 column (1.7 µm, 2.1 × 100 mm). The mobile phase consisted of solvent A (water containing 0.1% formic acid and 5 mm NH_4_OAc) and solvent B (acetonitrile containing 0.1% formic acid).

### 4.21 Construction of AAV‐Ripk3‐KD Mice

AAV6‐CMV‐ZsGreen and AAV6‐CMV‐Cre were constructed by Hanbio Biotechnology Co., Ltd. AAV was administered via intratracheal instillation, using the same instillation method as the BLM‐induced lung fibrosis model. Each mouse received 1 × 10^11^ genome copies of the virus. Two weeks post‐infection with AAV6‐CMV‐ZsGreen, the thoracic cavity was exposed. Mice were placed in the IVIS Spectrum bioluminescence imaging system for imaging, and the signal intensity of green fluorescence in the lungs was detected under 488 nm excitation light.

### 4.22 Reagents and Antibodies

A complete list of the reagents and antibodies used in this manuscript can be found in Table S2.

### 4.23 Statistical Analysis

All data in this study were derived from at least three independent biological replicates and are presented as mean ± standard error of the mean (SEM). Statistical analysis was performed using GraphPad Prism software. Overall differences in significance between groups were assessed using one‐way analysis of variance (ANOVA) or two‐way ANOVA. In specific instances, such as direct comparisons between two groups, unpaired two‐tailed Student's *t*‐tests were employed to determine statistically significant differences. The level of significance was set at *p* < 0.05; a *p*‐value less than 0.05 was considered to indicate a statistically significant difference.

## Author Contributions

T.Y. designed the experiments, did most of the experiments, compiled the data, prepared the figures, and wrote and revised the manuscript. X.L. performed bioinformatic analyses related to single‐cell transcriptomics data. S.L., Q.L., Z.H., J.R., X.Y., C.F., and Y.X. were involved in experiments related to the in vivo experiments. Y.Z. and X.Y. performed the experiments related to single‐cell sequencing. D.Z. supervised the single‐cell experiments and data analysis. W.T. conceived, supervised, and secured funding for the project. All authors provided feedback on the original and revised manuscript.

## Conflicts of Interest

The authors declare no conflicts of interest.

## Supporting information




**Supporting File**: advs74406‐sup‐0001‐SuppMat.docx.

## Data Availability

The data that support the findings of this study are available from the corresponding author upon reasonable request.

## References

[1] G. Raghu , S. Y. Chen , W. S. Yeh , et al., “Idiopathic Pulmonary Fibrosis in Us Medicare Beneficiaries Aged 65 Years and Older: Incidence, Prevalence, and Survival, 2001–11,” The Lancet Respiratory Medicine 2, no. 7 (2014): 566–572, 10.1016/s2213-2600(14)70101-8. 24875841

[2] T. M. Maher , S. Assassi , A. Azuma , et al., “Nerandomilast in Patients With Progressive Pulmonary Fibrosis,” New England Journal of Medicine 392, no. 22 (2025): 2203–2214, 10.1056/NEJMoa2503643. 40388329

[3] D. Reininger , K. Fundel‐Clemens , C. H. Mayr , et al., “Pde4b Inhibition by Nerandomilast: Effects on Lung Fibrosis and Transcriptome in Fibrotic Rats and on Biomarkers in Human Lung Epithelial Cells,” British Journal of Pharmacology 181, no. 23 (2024): 4766–4781, 10.1111/bph.17303. 39183442

[4] T. M. Maher , S. Assassi , A. Azuma , et al., “Design of a Phase Iii, Double‐Blind, Randomised, Placebo‐Controlled Trial of Bi 1015550 in Patients With Progressive Pulmonary Fibrosis (Fibroneer‐Ild),” BMJ Open Respiratory Research 10, no. 1 (2023): 001580, 10.1136/bmjresp-2022-001580. PMC10503394 37709661

[5] A. E. John , C. Joseph , G. Jenkins , and A. L. Tatler , “COVID‐19 and Pulmonary Fibrosis: A Potential Role for Lung Epithelial Cells and Fibroblasts,” Immunological Reviews 302, no. 1 (2021): 228–240, 10.1111/imr.12977. 34028807 PMC8237078

[6] A. Chakraborty , M. Mastalerz , M. Ansari , H. B. Schiller , and C. A. Staab‐Weijnitz , “Emerging Roles of Airway Epithelial Cells in Idiopathic Pulmonary Fibrosis,” Cells 11, no. 6 (2022): 1050, https://www.mdpi.com/2073‐4409/11/6/1050. 35326501 10.3390/cells11061050 PMC8947093

[7] P. Confalonieri , M. C. Volpe , J. Jacob , et al., “Regeneration or Repair? The Role of Alveolar Epithelial Cells in the Pathogenesis of Idiopathic Pulmonary Fibrosis (Ipf),” Cells 11, no. 13 (2022): 2095, https://www.mdpi.com/2073‐4409/11/13/2095. 35805179 10.3390/cells11132095 PMC9266271

[8] X. Di , J. Chen , Y. Li , et al., “Crosstalk Between Fibroblasts and Immunocytes in Fibrosis: From Molecular Mechanisms to Clinical Trials,” Clinical and Translational Medicine 14, no. 1 (2024): 1545, 10.1002/ctm2.1545. PMC10807359 38264932

[9] S. He , L. Wang , L. Miao , et al., “Receptor Interacting Protein Kinase‐3 Determines Cellular Necrotic Response to TNF‐α,” Cell 137, no. 6 (2009): 1100–1111, 10.1016/j.cell.2009.05.021. 19524512

[10] D. A. Rodriguez , R. Weinlich , S. Brown , et al., “Characterization of Ripk3‐Mediated Phosphorylation of the Activation Loop of Mlkl During Necroptosis,” Cell Death & Differentiation 23, no. 1 (2016): 76–88, 10.1038/cdd.2015.70. 26024392 PMC4815980

[11] K. Moriwaki and F. K. Chan , “The Inflammatory Signal Adaptor Ripk3: Functions Beyond Necroptosis,” International Review of Cell and Molecular Biology 328, no. (2017): 253–275, 10.1016/bs.ircmb.2016.08.007. 28069136 PMC5791152

[12] H. H. Park , H. R. Kim , S. Y. Park , et al., “Ripk3 Activation Induces Trim28 Derepression in Cancer Cells and Enhances the Anti‐Tumor Microenvironment,” Molecular Cancer 20, no. 1 (2021): 107, 10.1186/s12943-021-01399-3. 34419074 PMC8379748

[13] M. Oliver Metzig , Y. Tang , S. Mitchell , et al., “An Incoherent Feedforward Loop Interprets NFκB/RelA Dynamics to Determine TNF‐Induced Necroptosis Decisions,” Molecular Systems Biology 16, no. 12 (2020): 9677, 10.15252/msb.20209677. PMC7734648 33314666

[14] B. Yang , L. A. Maddison , K. E. Zaborska , et al., “Ripk3‐Mediated Inflammation Is a Conserved Β Cell Response to Er Stress,” Science Advances 6, no. 51 (2020): abd7272, 10.1126/sciadv.abd7272. PMC11206196 33355143

[15] M. Zheng , E. P. Williams , R. K. S. Malireddi , et al., “Impaired Nlrp3 Inflammasome Activation/Pyroptosis Leads to Robust Inflammatory Cell Death Via Caspase‐8/Ripk3 During Coronavirus Infection,” Journal of Biological Chemistry 295, no. 41 (2020): 14040–14052, 10.1074/jbc.RA120.015036. 32763970 PMC7549031

[16] X. Lei , Y. Chen , E. Lien , and K. A. Fitzgerald , “Mlkl‐Driven Inflammasome Activation and Caspase‐8 Mediate Inflammatory Cell Death in Influenza a Virus Infection, MBio ” 14, no. 2 (2023): 0011023, 10.1128/mbio.00110-23. PMC10127685 36852999

[17] K. E. Lawlor , N. Khan , A. Mildenhall , et al., “Ripk3 Promotes Cell Death and Nlrp3 Inflammasome Activation in the Absence of Mlkl,” Nature Communications 6, no. (2015): 6282, 10.1038/ncomms7282. PMC4346630 25693118

[18] S. Torii and S. Shimizu , “Involvement of Phosphorylation of Ulk1 in Alternative Autophagy,” Autophagy 16, no. 8 (2020): 1532–1533, 10.1080/15548627.2020.1776476. 32543339 PMC7469672

[19] S. Liu , K. Joshi , M. F. Denning , and J. Zhang , “Ripk3 Signaling and Its Role in the Pathogenesis of Cancers,” Cellular and Molecular Life Sciences 78, no. 23 (2021): 7199–7217, 10.1007/s00018-021-03947-y. 34654937 PMC9044760

[20] S. Torii , H. Yamaguchi , A. Nakanishi , et al., “Identification of a Phosphorylation Site on Ulk1 Required for Genotoxic Stress‐Induced Alternative Autophagy,” Nature Communications 11, no. 1 (2020): 1754, 10.1038/s41467-020-15577-2. PMC7145817 32273498

[21] M. J. Morgan and Y. S. Kim , “Roles of Ripk3 in Necroptosis, Cell Signaling, and Disease,” Experimental & Molecular Medicine 54, no. 10 (2022): 1695–1704, 10.1038/s12276-022-00868-z. 36224345 PMC9636380

[22] Z. Yang , Y. Wang , Y. Zhang , et al., “Rip3 Targets Pyruvate Dehydrogenase Complex to Increase Aerobic Respiration in Tnf‐Induced Necroptosis,” Nature Cell Biology 20, no. 2 (2018): 186–197, 10.1038/s41556-017-0022-y. 29358703

[23] D. W. Zhang , J. Shao , J. Lin , et al., “Rip3, an Energy Metabolism Regulator That Switches Tnf‐Induced Cell Death From Apoptosis to Necrosis,” Science 325, no. 5938 (2009): 332–336, 10.1126/science.1172308. 19498109

[24] L. Tao , Y. Yi , Y. Chen , et al., “Rip1 Kinase Activity Promotes Steatohepatitis Through Mediating Cell Death and Inflammation in Macrophages,” Cell Death & Differentiation 28, no. 4 (2021): 1418–1433, 10.1038/s41418-020-00668-w. 33208891 PMC8027792

[25] M. B. Afonso , P. M. Rodrigues , M. Mateus‐Pinheiro , et al., “Ripk3 Acts as a Lipid Metabolism Regulator Contributing to Inflammation and Carcinogenesis in Non‐Alcoholic Fatty Liver Disease,” Gut 70, no. 12 (2021): 2359–2372, 10.1136/gutjnl-2020-321767. 33361348 PMC8588316

[26] T. Wang , D. Wang , G. Kuang , et al., “Derlin‐1 Promotes Diet‐Induced Non‐Alcoholic Fatty Liver Disease Via Increasing Ripk3‐Mediated Necroptosis,” Free Radical Biology and Medicine 217, (2024): 29–47, 10.1016/j.freeradbiomed.2024.03.014. 38522486

[27] S. P. Preston , M. D. Stutz , C. C. Allison , et al., “Epigenetic Silencing of Ripk3 in Hepatocytes Prevents Mlkl‐Mediated Necroptosis From Contributing to Liver Pathologies,” Gastroenterology 163, no. 6 (2022): 1643–1657.e14, 10.1053/j.gastro.2022.08.040. 36037995

[28] M. B. Afonso , T. Islam , J. Magusto , et al., “Ripk3 Dampens Mitochondrial Bioenergetics and Lipid Droplet Dynamics in Metabolic Liver Disease,” Hepatology 77, no. 4 (2023): 1319–1334, 10.1002/hep.32756. 36029129 PMC10026966

[29] A. Majdi , L. Aoudjehane , V. Ratziu , et al., “Inhibition of Receptor‐Interacting Protein Kinase 1 Improves Experimental Non‐Alcoholic Fatty Liver Disease,” Journal of Hepatology 72, no. 4 (2020): 627–635, 10.1016/j.jhep.2019.11.008. 31760070

[30] H. Tye , S. A. Conos , T. M. Djajawi , et al., “Divergent Roles of RIPK3 and MLKL in High‐Fat Diet–Induced Obesity and MAFLD in Mice,” Life Science Alliance 8, no. 1 (2025): 202302446, 10.26508/lsa.202302446. PMC11557689 39532538

[31] S. Mohammed , N. Thadathil , P. Ohene‐Marfo , et al., “Absence of Either Ripk3 or Mlkl Reduces Incidence of Hepatocellular Carcinoma Independent of Liver Fibrosis,” Molecular Cancer Research 21, no. 9 (2023): 933–946, 10.1158/1541-7786.Mcr-22-0820. 37204757 PMC10472095

[32] J. M. Lee , M. Yoshida , M. S. Kim , et al., “Involvement of Alveolar Epithelial Cell Necroptosis in Idiopathic Pulmonary Fibrosis Pathogenesis,” American Journal of Respiratory Cell and Molecular Biology 59, no. 2 (2018): 215–224, 10.1165/rcmb.2017-0034OC. 29444413

[33] H. Chen , Z. Xia , B. Qing , et al., “Analysis of Necroptosis‐Related Prognostic Genes and Immune Infiltration in Idiopathic Pulmonary Fibrosis,” Frontiers in Immunology 14, no. (2023): 1119139, 10.3389/fimmu.2023.1119139. 37051233 PMC10083386

[34] M. Hao , X. Han , Z. Yao , et al., “The Pathogenesis of Organ Fibrosis: Focus on Necroptosis,” British Journal of Pharmacology 180, no. 22 (2023): 2862–2879, 10.1111/bph.15952. 36111431

[35] J. Guerrero‐Mauvecin , M. Fontecha‐Barriuso , A. M. López‐Diaz , A. Ortiz , and A. B. Sanz , “Ripk3 and Kidney Disease,” Nefrología 44, no. 1 (2024): 10–22, 10.1016/j.nefroe.2023.04.006. 37150671

[36] D. Martin‐Sanchez , J. Guerrero‐Mauvecin , M. Fontecha‐Barriuso , et al., “Bone Marrow–Derived RIPK3 Mediates Kidney Inflammation in Acute Kidney Injury,” Journal of the American Society of Nephrology 33, no. 2 (2022): 357–373, 10.1681/asn.2021030383. 35046131 PMC8819996

[37] A. Pefanis , A. K. Bongoni , J. L. McRae , et al., “Inhibition of Ripk1 or Ripk3 Kinase Activity Post Ischemia‐Reperfusion Reduces the Development of Chronic Kidney Injury,” Biochemical Journal 482, no. 2 (2025): 73–86, 10.1042/bcj20240569. 39705008 PMC12220529

[38] Y. Shi , X. Chen , C. Huang , and C. Pollock , “Ripk3: A New Player in Renal Fibrosis,” Frontiers in Cell and Developmental Biology 8, no. (2020): 502, 10.3389/fcell.2020.00502. 32613000 PMC7308494

[39] Y. J. Jiang , J. Jin , Q. Y. Nan , et al., “Coenzyme Q10 Attenuates Renal Fibrosis by Inhibiting Rip1‐Rip3‐Mlkl‐Mediated Necroinflammation Via Wnt3α/Β‐Catenin/Gsk‐3β Signaling in Unilateral Ureteral Obstruction,” International Immunopharmacology 108, no. (2022): 108868, 10.1016/j.intimp.2022.108868. 35636077

[40] Y. Wang , L. Yu , Y. Li , et al., “Supplemented Gegen Qinlian Decoction Formula Attenuates Podocyte Mitochondrial Fission and Renal Fibrosis in Diabetic Kidney Disease by Inhibiting Tnf‐Α‐Mediated Necroptosis, Compared With Empagliflozin,” Journal of Ethnopharmacology 334, no. (2024): 118572, 10.1016/j.jep.2024.118572. 39025164

[41] M. A. Abou Taha , F. E. M. Ali , I. G. Saleh , and E. S. Akool , “Sorafenib and Edaravone Protect Against Renal Fibrosis Induced by Unilateral Ureteral Obstruction Via Inhibition of Oxidative Stress, Inflammation, and Ripk‐3/Mlkl Pathway,” Naunyn‐Schmiedeberg's Archives of Pharmacology 397, no. 11 (2024): 8961–8977, 10.1007/s00210-024-03146-z. 38874805 PMC11522075

[42] S. G. Piao , J. Ding , X. J. Lin , et al., “RETRACTED: Inhibition of RIP1‐RIP3‐mediated necroptosis attenuates renal fibrosis via Wnt3α/β‐catenin/GSK‐3β signaling in unilateral ureteral obstruction,” PLoS ONE 17, no. 10 (2022): 0274116, 10.1371/journal.pone.0274116. PMC9555645 36223414

[43] L. C. Davies , S. J. Jenkins , J. E. Allen , and P. R. Taylor , “Tissue‐Resident Macrophages,” Nature Immunology 14, no. 10 (2013): 986–995, 10.1038/ni.2705. 24048120 PMC4045180

[44] H. Aegerter , B. N. Lambrecht , and C. V. Jakubzick , “Biology of Lung Macrophages in Health and Disease,” Immunity 55, no. 9 (2022): 1564–1580, 10.1016/j.immuni.2022.08.010. 36103853 PMC9533769

[45] M. Guilliams , G. R. Thierry , J. Bonnardel , and M. Bajenoff , “Establishment and Maintenance of the Macrophage Niche,” Immunity 52, no. 3 (2020): 434–451, 10.1016/j.immuni.2020.02.015. 32187515

[46] M. Guilliams , I. De Kleer , S. Henri , et al., “Alveolar Macrophages Develop From Fetal Monocytes That Differentiate Into Long‐Lived Cells in the First Week of Life Via Gm‐Csf,” Journal of Experimental Medicine 210, no. 10 (2013): 1977–1992, 10.1084/jem.20131199. 24043763 PMC3782041

[47] T. Fabre , A. M. S. Barron , S. M. Christensen , et al., “Identification of a Broadly Fibrogenic Macrophage Subset Induced by Type 3 Inflammation,” Science Immunology 8, no. 82 (2023): add8945, 10.1126/sciimmunol.add8945. 37027478

[48] M. Cruz Tleugabulova , S. P. Melo , A. Wong , et al., “Induction of a Distinct Macrophage Population and Protection From Lung Injury and Fibrosis by Notch2 Blockade,” Nature Communications 15, no. 1 (2024): 9575, 10.1038/s41467-024-53700-9. PMC11541919 39505846

[49] H. Han , X. Ge , S. Santosh Babu Komakula , et al., “Macrophage‐Derived Osteopontin (Spp1) Protects From Nonalcoholic Steatohepatitis,” Gastroenterology 165, no. 1 (2023): 201–217, 10.1053/j.gastro.2023.03.228. 37028770 PMC10986640

[50] C. Ruscitti , J. Abinet , P. Maréchal , et al., “Recruited Atypical Ly6g^+^ Macrophages License Alveolar Regeneration After Lung Injury,” Science Immunology 9, no. 98 (2024): ado1227, 10.1126/sciimmunol.ado1227. PMC7616420 39093958

[51] K. Hoeft , G. J. L. Schaefer , H. Kim , et al., “Platelet‐Instructed Spp^1+^ Macrophages Drive Myofibroblast Activation in Fibrosis in a Cxcl4‐Dependent Manner,” Cell Reports 42, no. 2 (2023): 112131, 10.1016/j.celrep.2023.112131. 36807143 PMC9992450

[52] T. S. Adams , J. C. Schupp , S. Poli , et al., “Single‐Cell Rna‐Seq Reveals Ectopic and Aberrant Lung‐Resident Cell Populations in Idiopathic Pulmonary Fibrosis,” Science Advances 6, no. 28 (2020): aba1983, 10.1126/sciadv.aba1983. PMC7439502 32832599

[53] C. Morse , T. Tabib , J. Sembrat , et al., “Proliferating Spp1/Mertk‐Expressing Macrophages in Idiopathic Pulmonary Fibrosis,” European Respiratory Journal 54, no. 2 (2019): 1802441, 10.1183/13993003.02441-2018. 31221805 PMC8025672

[54] P. A. Reyfman , J. M. Walter , N. Joshi , et al., “Single‐Cell Transcriptomic Analysis of Human Lung Provides Insights Into the Pathobiology of Pulmonary Fibrosis,” American Journal of Respiratory and Critical Care Medicine 199, no. 12 (2019): 1517–1536, 10.1164/rccm.201712-2410OC. 30554520 PMC6580683

[55] A. V. Misharin , L. Morales‐Nebreda , P. A. Reyfman , et al., “Monocyte‐Derived Alveolar Macrophages Drive Lung Fibrosis and Persist in the Lung Over the Life Span,” Journal of Experimental Medicine 214, no. 8 (2017): 2387–2404, 10.1084/jem.20162152. 28694385 PMC5551573

[56] Y. Wang , Q. Wang , S. Tao , et al., “Identification of Spp1+ Macrophages in Promoting Cancer Stemness Via Vitronectin and Ccl15 Signals Crosstalk in Liver Cancer,” Cancer Letters 604, (2024): 217199, 10.1016/j.canlet.2024.217199. 39216547

[57] M. Shan , X. Yuan , L. Song , et al., “Cigarette Smoke Induction of Osteopontin (Spp1) Mediates Th17 Inflammation in Human and Experimental Emphysema,” Science Translational Medicine 4, no. 117 (2012): 117ra119, 10.1126/scitranslmed.3003041. PMC3956594 22261033

[58] E. Matsubara , Y. Komohara , S. Esumi , et al., “Spp1 Derived From Macrophages Is Associated With a Worse Clinical Course and Chemo‐Resistance in Lung Adenocarcinoma,” Cancers 14, no. 18 (2022): 4374, 10.3390/cancers14184374. 36139536 PMC9496817

[59] X. Yu , A. Buttgereit , I. Lelios , et al., “The Cytokine TGF‐β Promotes the Development and Homeostasis of Alveolar Macrophages,” Immunity 47, no. 5 (2017): 903–912.e4, 10.1016/j.immuni.2017.10.007. 29126797

[60] S. Chen , D. Luo , W. J. Streit , and J. K. Harrison , “Tgf‐Β1 Upregulates Cx3cr1 Expression and Inhibits Fractalkine‐Stimulated Signaling in Rat Microglia,” Journal of Neuroimmunology 133, no. 1 (2002): 46–55, 10.1016/S0165-5728(02)00354-5. 12446007

[61] M. Tardelli , K. Zeyda , V. Moreno‐Viedma , et al., “Osteopontin Is a Key Player for Local Adipose Tissue Macrophage Proliferation in Obesity,” Molecular Metabolism 5, no. 11 (2016): 1131–1137, 10.1016/j.molmet.2016.09.003. 27818939 PMC5081407

[62] A. Yim , C. Smith , and A. M. Brown , “Osteopontin/Secreted Phosphoprotein‐1 Harnesses Glial‐, Immune‐, and Neuronal Cell Ligand‐Receptor Interactions to Sense and Regulate Acute and Chronic Neuroinflammation,” Immunological Reviews 311, no. 1 (2022): 224–233, 10.1111/imr.13081. 35451082 PMC9790650

[63] A. L. McCubbrey , A. Shannon , J. D. McManus et al., “Polyamine Import and Accumulation Causes Immunomodulation in Macrophages Engulfing Apoptotic Cells,” Cell Reports 38, no. 2 (2022): 110222, 10.1016/j.celrep.2021.110222. 35021097 PMC8859864

[64] Z. Li , L. Wang , Y. Ren , et al., “Arginase: Shedding Light on the Mechanisms and Opportunities in Cardiovascular Diseases,” Cell Death Discovery 8, no. 1 (2022): 413, 10.1038/s41420-022-01200-4. 36209203 PMC9547100

[65] S. Cane , R. Geiger , and V. Bronte , “The Roles of Arginases and Arginine in Immunity,” Nature Reviews Immunology 25, no. 4 (2024): 266–284, 10.1038/s41577-024-01098-2. 39420221

[66] V. Bronte and P. Zanovello , “Regulation of Immune Responses by L‐Arginine Metabolism,” Nature Reviews Immunology 5, no. 8 (2005): 641–654, 10.1038/nri1668. 16056256

[67] A. Vannan , R. Lyu , A. L. Williams , et al., “Spatial Transcriptomics Identifies Molecular Niche Dysregulation Associated With Distal Lung Remodeling in Pulmonary Fibrosis,” Nature Genetics 57, no. 3 (2025): 647–658, 10.1038/s41588-025-02080-x. 39901013 PMC11906353

[68] C. H. Mayr , D. Santacruz , S. Jarosch , et al., “Spatial Transcriptomic Characterization of Pathologic Niches in Ipf,” Science Advances 10, no. 32 (2024): adl5473, 10.1126/sciadv.adl5473. PMC11313858 39121212

[69] L. Franzén , M. Olsson Lindvall , M. Hühn , et al., “Mapping Spatially Resolved Transcriptomes in Human and Mouse Pulmonary Fibrosis,” Nature Genetics 56, no. 8 (2024): 1725–1736, 10.1038/s41588-024-01819-2. 38951642 PMC11319205

[70] Y. Gu , T. Lawrence , R. Mohamed , Y. Liang , and B. H. Yahaya , “The Emerging Roles of Interstitial Macrophages in Pulmonary Fibrosis: A Perspective From Scrna‐Seq Analyses,” Frontiers in Immunology 13, no. (2022): 923235, 10.3389/fimmu.2022.923235. 36211428 PMC9536737

[71] J. Li , X. Zhai , X. Sun , S. Cao , Q. Yuan , and J. Wang , “Metabolic Reprogramming of Pulmonary Fibrosis,” Frontiers in Pharmacology 13, no. (2022): 1031890, 10.3389/fphar.2022.1031890. 36452229 PMC9702072

[72] L. Yan , J. Wang , X. Cai , et al., “Macrophage Plasticity: Signaling Pathways, Tissue Repair, and Regeneration,” MedComm 5, no. 8 (2024): 658, 10.1002/mco2.658. PMC11292402 39092292

[73] C. Jing , T. Castro‐Dopico , N. Richoz , et al., “Macrophage Metabolic Reprogramming Presents a Therapeutic Target in Lupus Nephritis,” Proceedings of the National Academy of Sciences 117, no. 26 (2020): 15160–15171, 10.1073/pnas.2000943117. PMC7334513 32541026

[74] Z. Wang , F. Zhao , C. Xu , et al., “Metabolic Reprogramming in Skin Wound Healing,” Burns & Trauma 12, (2024): tkad047, 10.1093/burnst/tkad047. 38179472 PMC10762507

[75] A. Viola , F. Munari , R. Sánchez‐Rodríguez , T. Scolaro , and A. Castegna , “The Metabolic Signature of Macrophage Responses,” Frontiers in Immunology 10, (2019): 1462, 10.3389/fimmu.2019.01462. 31333642 PMC6618143

[76] A. Yurdagul , M. Subramanian , X. Wang , et al., “Macrophage Metabolism of Apoptotic Cell‐Derived Arginine Promotes Continual Efferocytosis and Resolution of Injury,” Cell Metabolism 31, no. 3 (2020): 518–533.e10, 10.1016/j.cmet.2020.01.001. 32004476 PMC7173557

[77] L. A. Monticelli , M. D. Buck , A. L. Flamar , et al., “Arginase 1 Is an Innate Lymphoid‐Cell‐Intrinsic Metabolic Checkpoint Controlling Type 2 Inflammation,” Nature Immunology 17, no. 6 (2016): 656–665, 10.1038/ni.3421. 27043409 PMC4873382

[78] R. Li , L. Yan , K. Jiang , et al., “Lighting up Arginine Metabolism Reveals Its Functional Diversity in Physiology and Pathology,” Cell Metabolism 37, no. 1 (2024): 291–304, 10.1016/j.cmet.2024.09.011. 39413790

[79] W. Durante , L. Liao , S. V. Reyna , K. J. Peyton , and A. I. Schafer , “Transforming Growth Factor‐β 1 Stimulates l ‐Arginine Transport and Metabolism in Vascular Smooth Muscle Cells,” Circulation 103, no. 8 (2001): 1121–1127, 10.1161/01.cir.103.8.1121. 11222476

[80] L. J. Ignarro , G. M. Buga , L. H. Wei , P. M. Bauer , G. Wu , and P. Soldato , “Role of the Arginine‐Nitric Oxide Pathway in the Regulation of Vascular Smooth Muscle Cell Proliferation,” Proceedings of the National Academy of Sciences 98, no. 7 (2001): 4202–4208, 10.1073/pnas.071054698. PMC31203 11259671

[81] K. A. Niese , M. G. Chiaramonte , L. G. Ellies , M. E. Rothenberg , and N. Zimmermann , “The Cationic Amino Acid Transporter 2 Is Induced in Inflammatory Lung Models and Regulates Lung Fibrosis,” Respiratory Research 11, no. 1 (2010): 87, 10.1186/1465-9921-11-87. 20576117 PMC2906447

[82] M. Imamura , J. S. Moon , K. P. Chung , et al., “Ripk3 Promotes Kidney Fibrosis Via Akt‐Dependent Atp Citrate Lyase,” JCI Insight 3, no. 3 (2018), 10.1172/jci.insight.94979. PMC5821177 29415885

[83] R. M. Tighe , J. Liang , N. Liu , et al., “Recruited Exudative Macrophages Selectively Produce Cxcl10 After Noninfectious Lung Injury,” American Journal of Respiratory Cell and Molecular Biology 45, no. 4 (2011): 781–788, 10.1165/rcmb.2010-0471OC. 21330464 PMC3208617

[84] J. G. McComb , M. Ranganathan , X. H. Liu , et al., “Cx3cl1 up‐Regulation Is Associated With Recruitment of Cx3cr^1+^ Mononuclear Phagocytes and T Lymphocytes in the Lungs During Cigarette Smoke‐Induced Emphysema,” The American Journal of Pathology 173, no. 4 (2008): 949–961, 10.2353/ajpath.2008.071034. 18772344 PMC2543064

[85] B. K. Martin , C. Qiu , E. Nichols , et al., “Optimized Single‐Nucleus Transcriptional Profiling by Combinatorial Indexing,” Nature Protocols 18, no. 1 (2023): 188–207, 10.1038/s41596-022-00752-0. 36261634 PMC9839601

[86] R. Fuentes‐Mateos , E. Santos , and A. Fernández‐Medarde , “Optimized Protocol for Isolation and Culture of Murine Neonatal Primary Lung Fibroblasts,” Methods and Protocols 6, no. 1 (2023): 14, 10.3390/mps6010014. 36827501 PMC9966303

