# Supplementary material for: RIPK3 Orchestrates Scar‐Associated Macrophage Dysfunction to Drive Pulmonary Fibrosis
Source: Adv Sci (Weinh). 2026 Feb 19;13(23):e19404. doi: 10.1002/advs.202519404 (PMC13104126; doi:10.1002/advs.202519404)
Supplement: Supplementary file 1 — Supporting File: advs74406‐sup‐0001‐SuppMat.docx. [file ADVS-13-e19404-s001.docx]

**Supporting Information for**

RIPK3 Orchestrates Scar-Associated Macrophage Dysfunction to Drive Pulmonary Fibrosis

**Figures. S1-S10**

**Tables S1-S2**

**References**


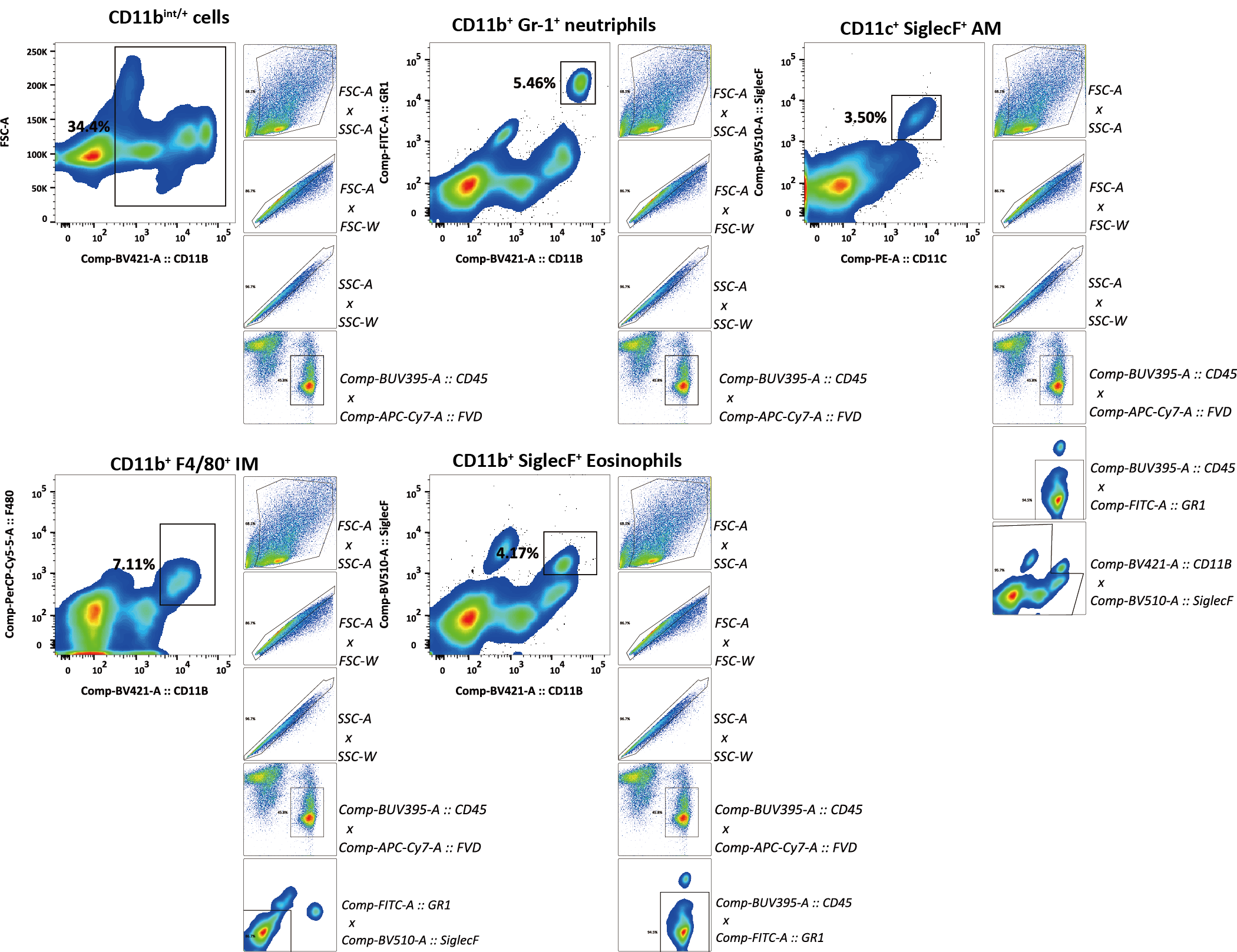


**Figure S1. Flow cytometry gating strategy.** Representative flow cytometry gating strategy showing live and single CD45^+^CD11b^int/+^ cells, CD45^+^CD11b^+^Gr-1^+^ neutrophils, CD45^+^Gr-1^-^CD11b^int/+^CD11C^+^SiglecF^+^ AMs, CD45^+^Gr-1^-^SiglecF^-^CD11b^+^F4/80^+^ IMs, and CD45^+^Gr-1^-^CD11b^+^ SiglecF^+^ eosinophils.


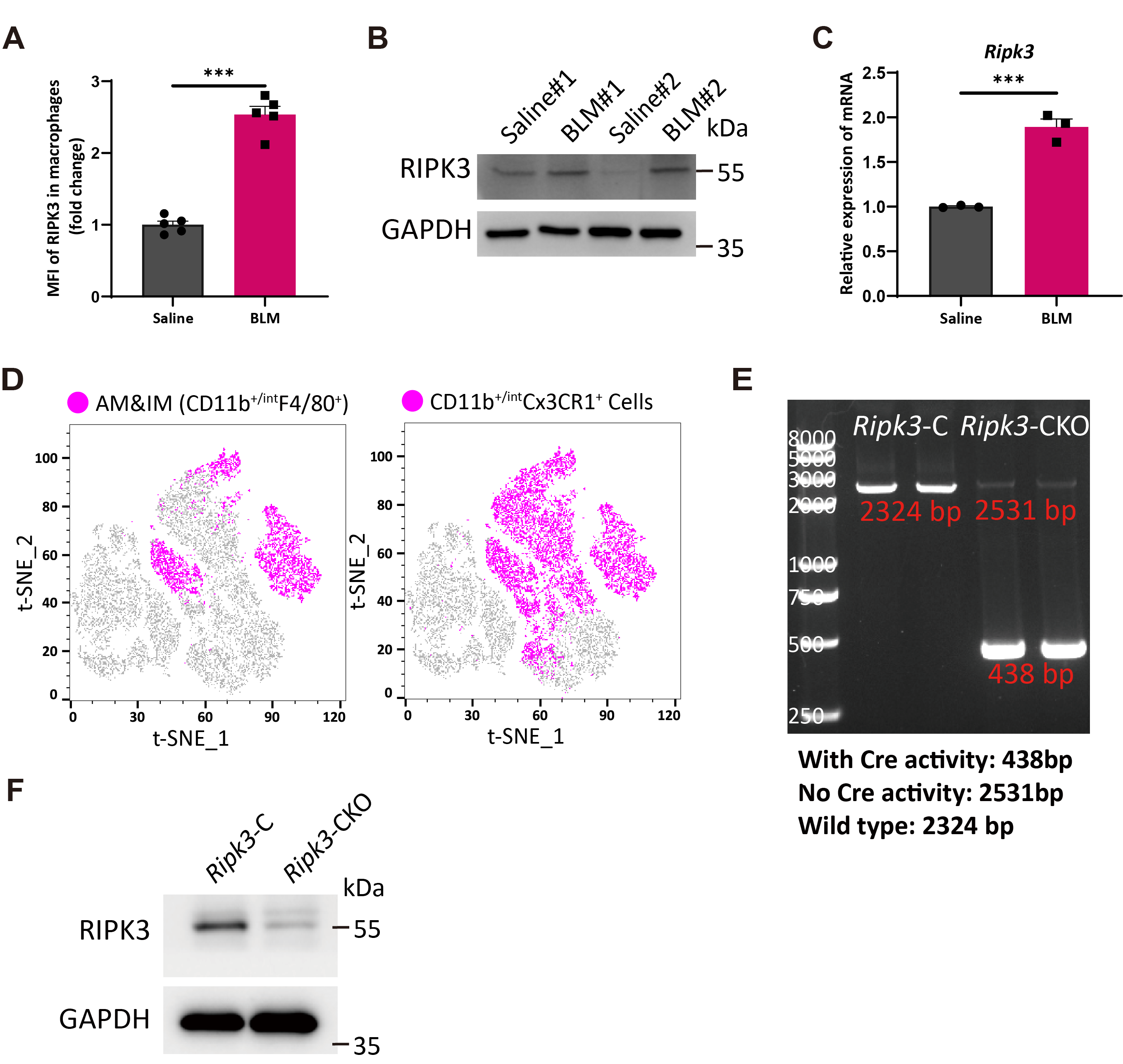


**Figure S2. RIPK3 increase in a mouse model of BLM-induced pulmonary fibrosis and establishment of *Ripk3*-CKO mice. (A**) Quantification of the mean fluorescence intensity of RIPK3 within F4/80^+^ areas by Image J. **(B)** Western Blot analysis of RIPK3 in the F4/80^+^ macrophages of lungs. **(C)** RT-qPCR analysis of *Ripk3* in the F4/80^+^ macrophages of lungs. Data show means ± SEM. **(D)** Representative t-SNE flow cytometry analysis plots of CD11b^int/+^ cells in lung tissue and the proportion of these cells among total lung single cells. **(E)** PCR identification of gene recombination in lung tissue. **(F)** Western Blot analysis of RIPK3 protein expression levels in BMDMs from *Ripk3*-C and *Ripk3*-CKO mice. (A) Symbols on bar graphs represent individual area. (C) Symbols on bar graphs represent F4/80^+^ macrophages from two mice. [(A), (C)] Student’s *t* tests was used. ^***^*P*<0.001 compared with the Saline group.


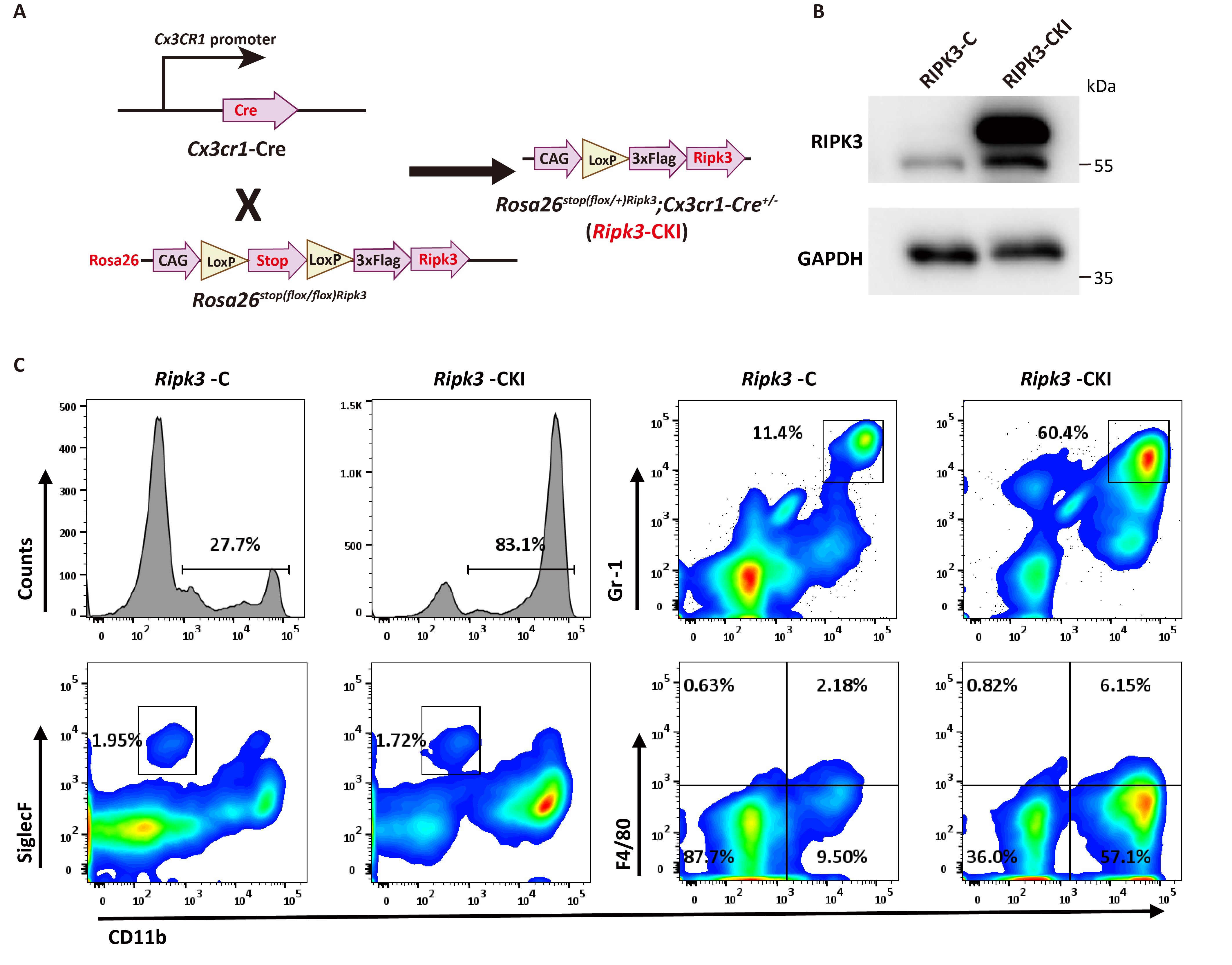


**Figure S3. Conditional knock-in of RIPK3 in macrophages caused dysplasia and lung inflammation in mice.** **(A)** Schematic diagram illustrating the construction of pulmonary macrophage-specific RIPK3 knock-in (*Ripk3*-CKI) mice. **(B)** Western Blot analysis of RIPK3 protein expression levels in peritoneal macrophages from *Ripk3*-C and *Ripk3*-CKI mice. **(C)** Representative flow cytometry analysis plots of myeloid cells (CD11b^+^), neutrophils (CD11b^+^Gr1^+^), alveolar macrophages (CD11b^int/+^SiglecF^+^), and interstitial macrophages (CD11b^+^F4/80^+^) in mouse lung tissue.


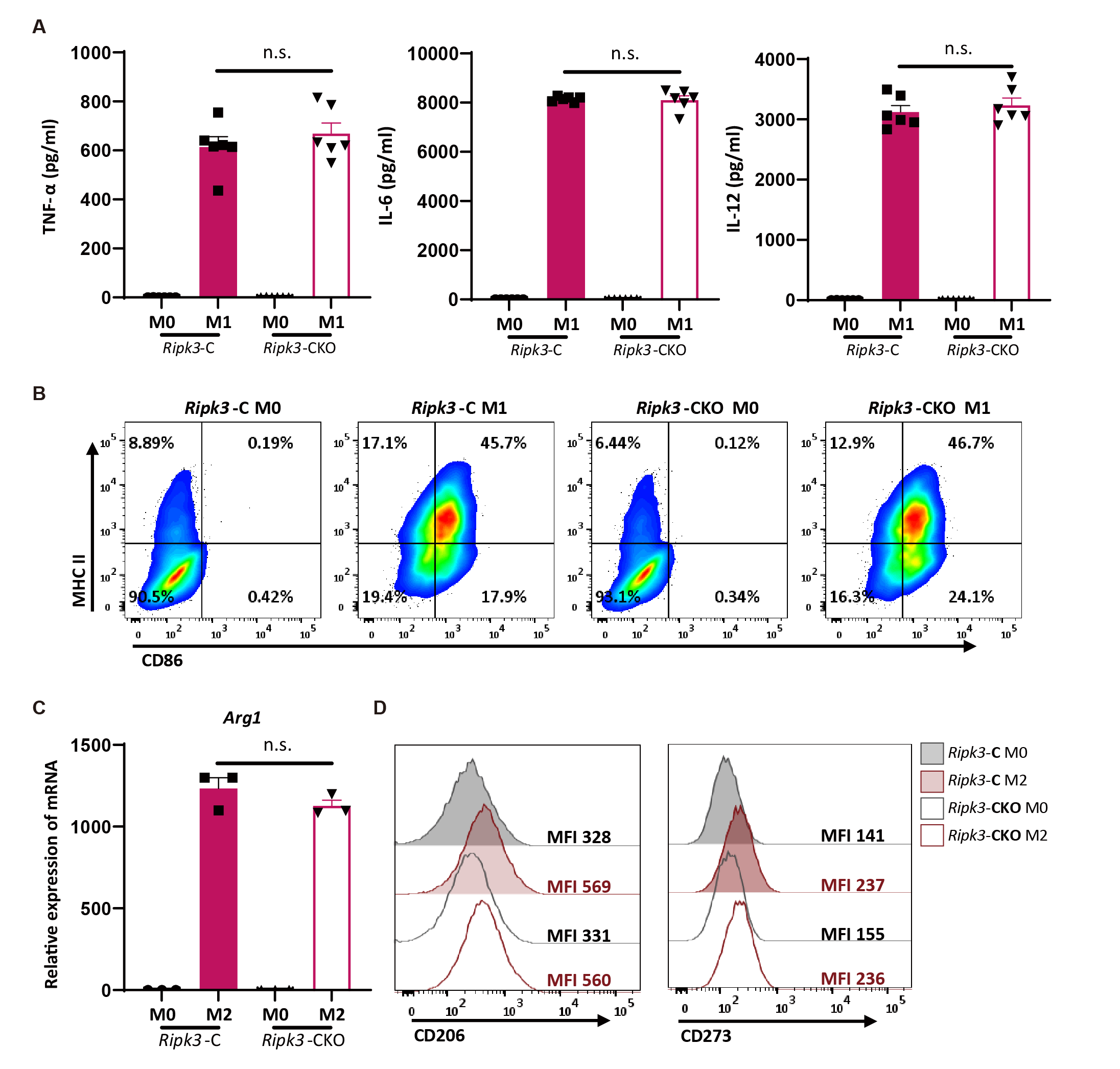


**Figure S4. RIPK3 knockdown did not inhibit macrophage M1/M2 polarization.** **(A)** Levels of cytokines TNF-α, IL-6, and IL-12 in supernatants of BMDMs stimulated with LPS (500 ng/mL) and IFN-γ (100 ng/mL) (M1 polarization), detected by ELISA. **(B)** Expression of CD86 and MHC II on M1-BMDMs (gated on CD11b^+^F4/80^+^ cells). **(C)** Gene expression of *Arg1* in BMDMs stimulated with IL-4 (20 ng/mL) and IL-13 (20 ng/mL) (M2 polarization), detected by RT-qPCR. **(D)** Expression of CD206 and CD273 on M2- BMDMs (gated on CD11b^+^F4/80^+^ cells). [(A), (C)] Data show means ± SEM and are representative of at least three independent experiments. Symbols on bar graphs represent independent experiments. [(A), (C)] one-way ANOVA with Dunnett’s multiple comparisons test was used. n.s., not significant; MFI, mean fluorescence intensity.


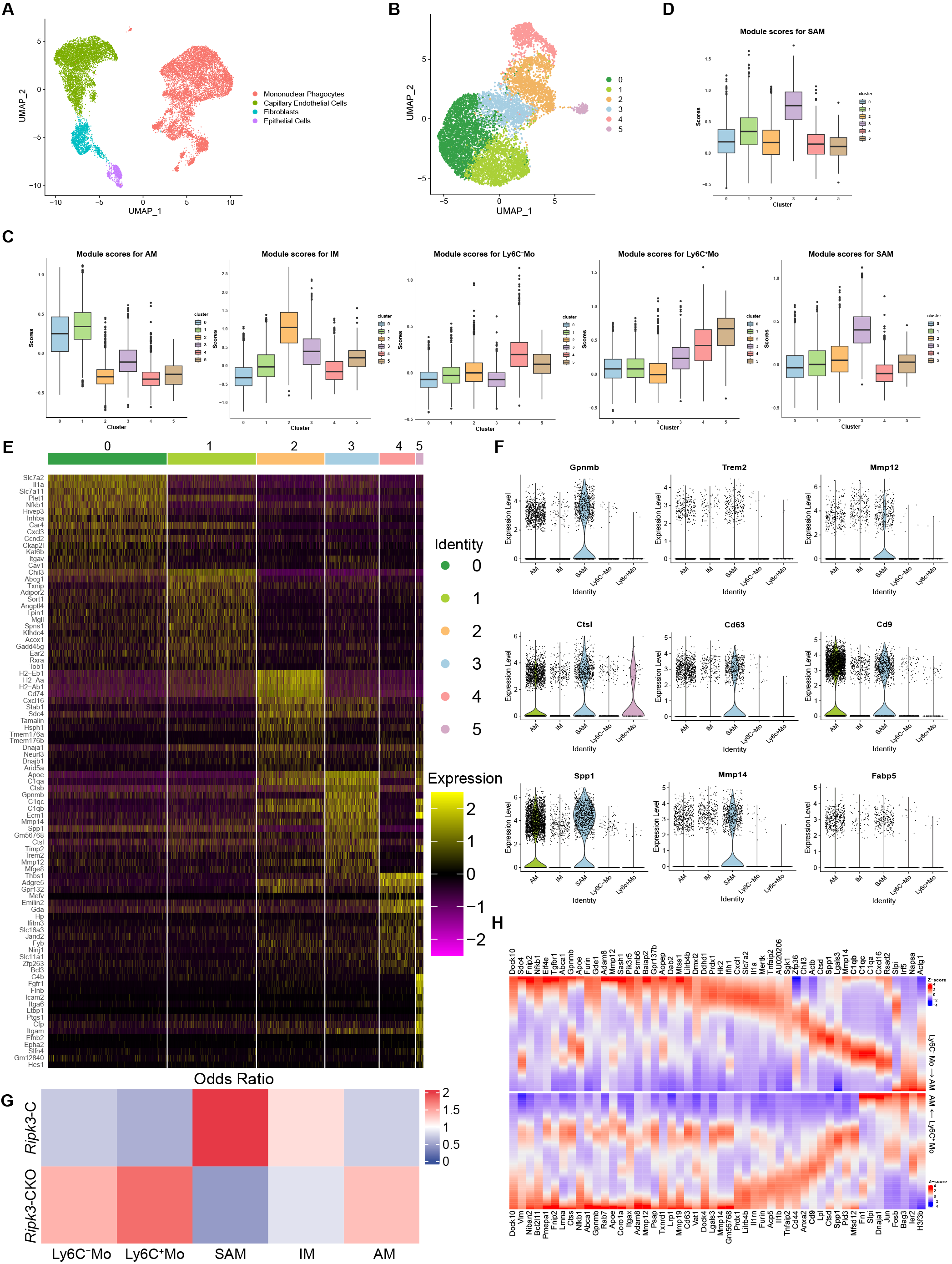


**Figure S5. Gene expression and features of clusters identified by scRNA-seq. (A)** UMAP plots of scRNA-seq data depicting the transcriptional identity of cell clusters sorted by F4/80 magnetic beads. **(B)** UMAP plots of mononuclear phagocytes from cell clusters sorted by F4/80 magnetic beads labeled by cell type. **(C)** Box plot showing the enrichment results of marker genes from the unknown cluster against reference cell type marker gene sets(1). **(D)** Box plot showing the enrichment results of marker genes from the unknown cluster against reference SAMs marker gene sets(2). **(E)** Heatmap showing the expression of the top 15 differentially expressed genes (DEGs) among different macrophage subpopulations. **(F)** Expression of SAM-associated marker genes in each macrophage subpopulation. **(G)** Heatmap of odds ratios for macrophage subtypes in the lungs of *Ripk3*-CKO versus *Ripk3*-C Mice. **(H)** Heatmap plot depicting the differentially expressed genes along pseudotime evaluated by tradeSeq in the trajectory starting from Ly6C^-^ Mo and Ly6C^+^ Mo ending in AMs.


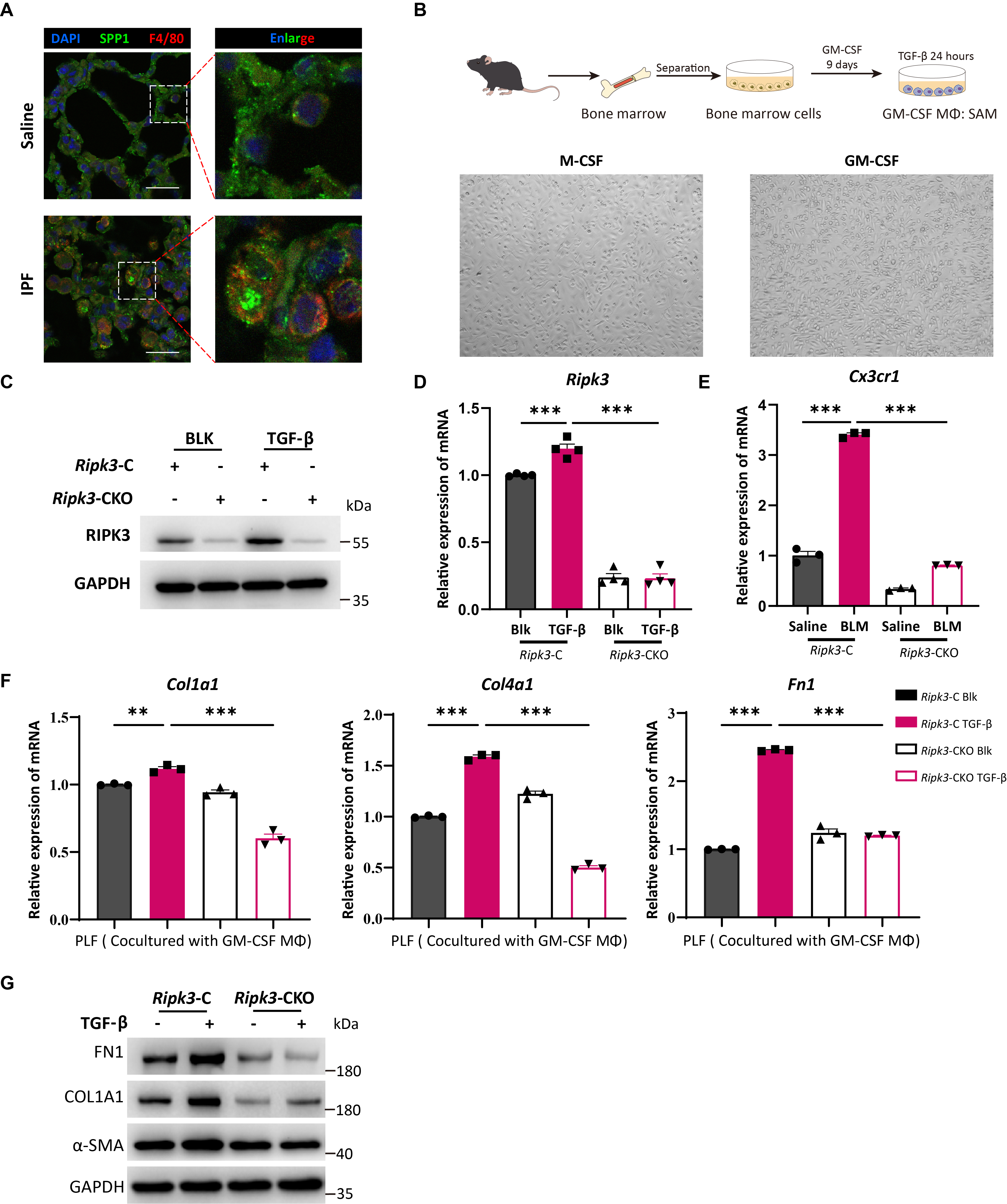


**Figure S6. *In vitro* induction process of SAM and evaluation of its pro-fibrotic function. (A)** Representative immunofluorescence images of SPP1 and F4/80 in lung tissue. Scale bar: 20 μm. **(B)** Schematic diagram of SAMs-induced differentiation and comparison with M-CSF-induced BMDM. **(C)** Western Blot analysis of RIPK3 expression levels in SAMs from *Ripk3*-C and *Ripk3*-CKO mice. **(D-E)** Gene expression of *Ripk3* and *Cx3cr1* in SAMs, detected by RT-qPCR. **(F)** RT-qPCR analysis of *Col1a1*, *Fn1*, and *Col4a1* gene expression in primary lung fibroblasts. **(G)** Western Blot analysis of FN1, COL1A1 and α-SMA expression levels in primary lung fibroblasts. [(D-F)] Data show means ± SEM and are representative of at least three independent experiments. Symbols on bar graphs represent independent experiments. [(D-F)] one-way ANOVA with Dunnett’s multiple comparisons test was used. ^**^*P*<0.01, ^***^*P*<0.001 compared with the indicated groups.


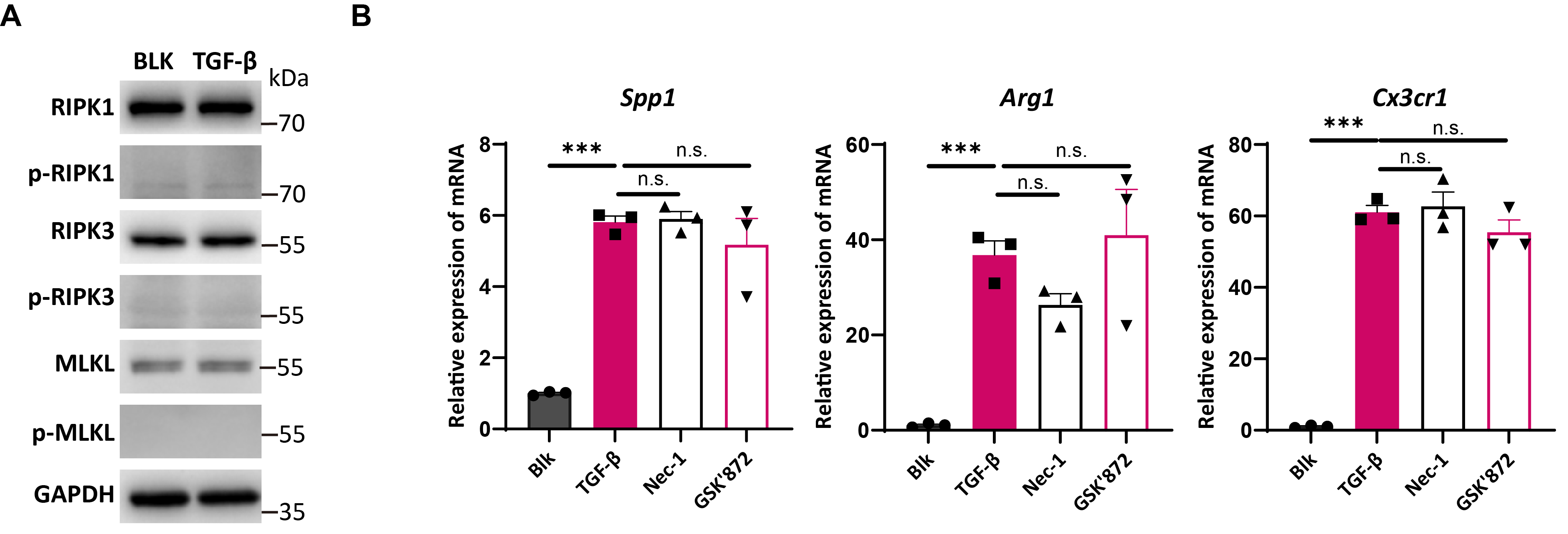


**Figure S7: RIPK3 regulates macrophage metabolic reprogramming via a necroptosis-independent mechanism.** **(A)** Western blot analysis of canonical necroptosis signaling markers in SAMs following stimulation with TGF-β for 8 hours. **(B)** RT-qPCR analysis of *Spp1*, *Arg1*, and *Cx3cr1* gene expression in SAMs. RIPK1 inhibitor Nec-1s (1 μM), or the RIPK3 kinase inhibitor GSK'872 (1 μM), followed by stimulation with TGF-β for 24 hours. (B) Data show means ± SEM and are representative of at least three independent experiments. Symbols on bar graphs represent independent experiments. (B) one-way ANOVA with Dunnett’s multiple comparisons test was used. ^***^*P*<0.001, n.s., no significance, compared with the indicated groups.


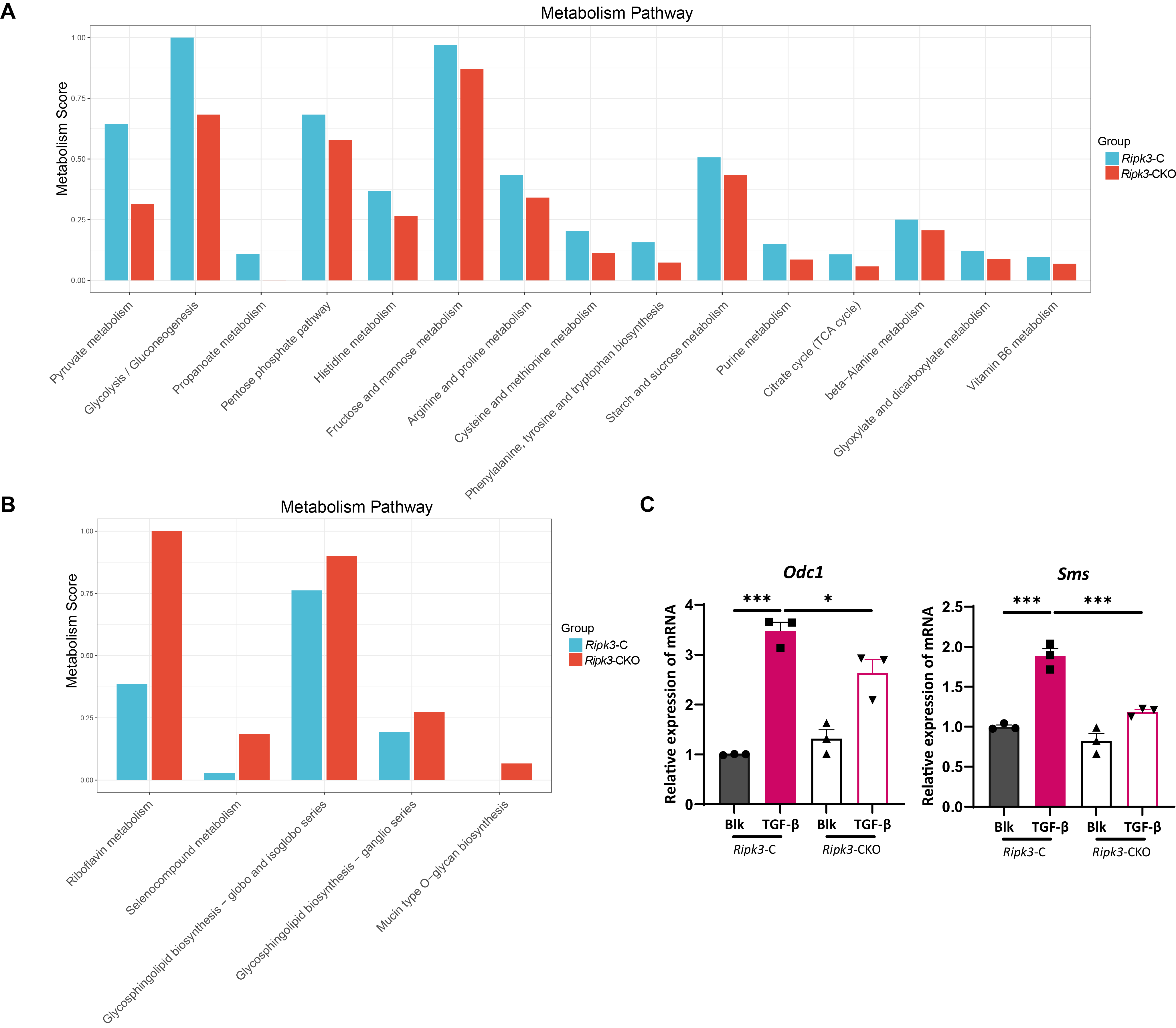


**Figure S8.** **Evaluation of metabolic pathways in SAMs. (A-B)** Standardized metabolic activity scores were calculated to compare RIPK3 knockout and control SAMs using the scMetabolism in IPF mice. **(C)** RT-qPCR analysis of *Odc1* and *Sms* mRNA expression in *Ripk3*-C and *Ripk3*-CKO SAMs treated with TGF-β1 for 24 hours. Data show means ± SEM and are representative of at least three independent experiments. Symbols on bar graphs represent independent experiments. (C) one-way ANOVA with Dunnett’s multiple comparisons test was used. ^*^*P*<0.05 and ^***^*P*<0.01, compared with the indicated groups.


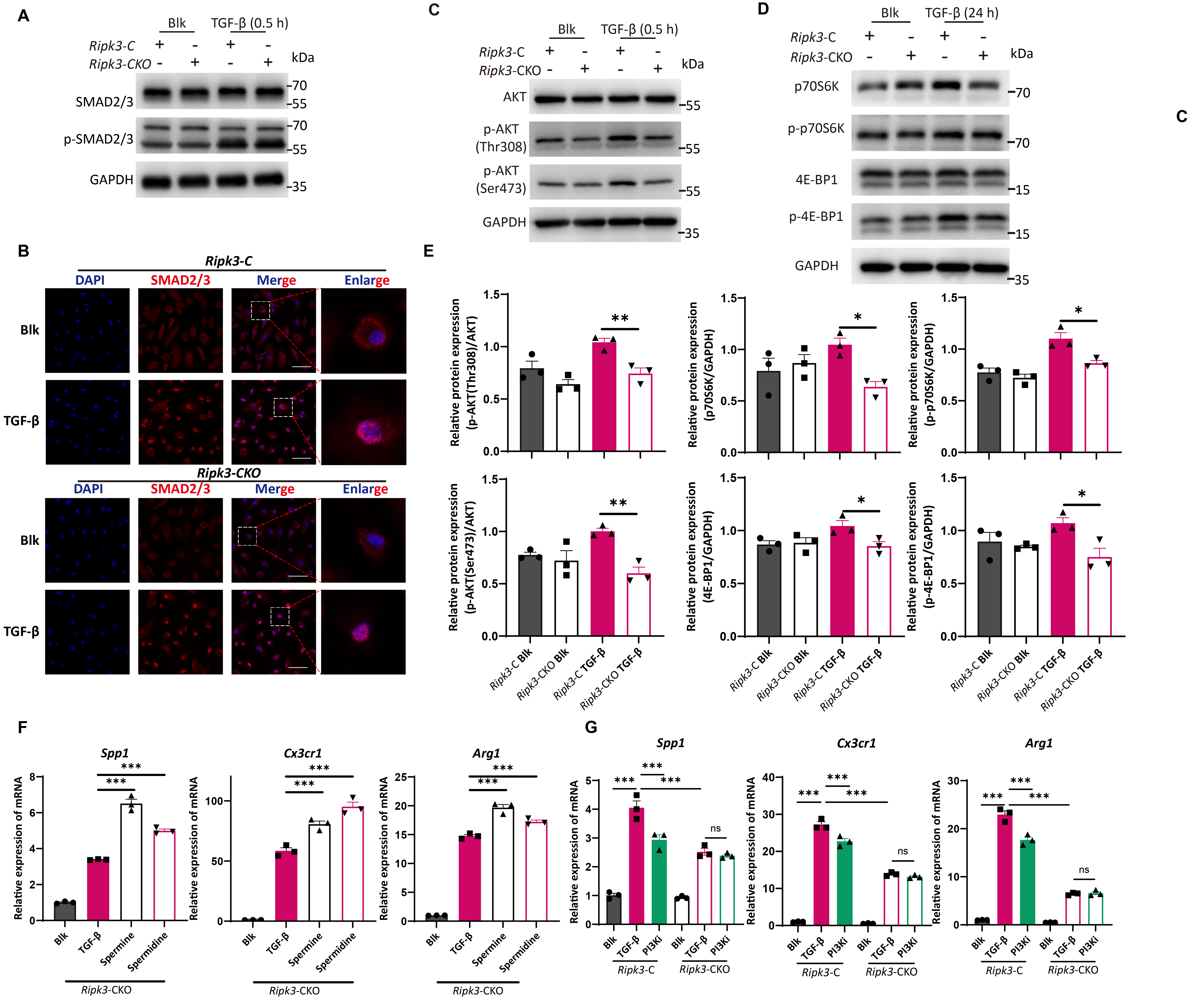


**Figure S9. RIPK3 drives Smad-independent TGF-β signaling through the PI3K-AKT-mTOR pathway.** **(A)** Western Blot analysis of intracellular levels of SMAD2/3 and p-SMAD2/3 in SAMs from *Ripk3*-C and *Ripk3*-CKO mice 0.5 hour after TGF-β stimulation. **(B)** Immunofluorescence detection of the cellular localization of intracellular SMAD2/3. Scale bar: 20 μm. **(C-D)** Western Blot analysis of intracellular levels of related proteins in SAMs from *Ripk3*-C and *Ripk3*-CKO mice at 0.5 and 24 hours after TGF-β stimulation. **(E)** Western Blot quantification of protein levels in SAMs after TGF-β stimulation in Fig. S8C-D. **(F)** RT-qPCR analysis of *Spp1*, *Arg1*, and *Cx3cr1* mRNA expression in *Ripk3*-C and *Ripk3*-CKO SAMs stimulated with TGF-β1 (10 ng/mL) in the presence or absence of exogenous spermine and spermidine (10 μM) for 24 hours. **(G)** RT-qPCR analysis of *Spp1*, *Arg1*, and *Cx3cr1* mRNA expression in *Ripk3*-C and *Ripk3*-CKO SAMs treated with TGF-β1 in the presence or absence of the PI3K inhibitor Omipalisib (10 nM) for 24 hours. Data show means ± SEM and are representative of at least three independent experiments. Symbols on bar graphs represent independent experiments. (E-G) one-way ANOVA with Dunnett’s multiple comparisons test was used. ^*^*P*<0.05, ^**^*P*<0.01 and ^***^*P*<0.01, compared with the indicated groups.


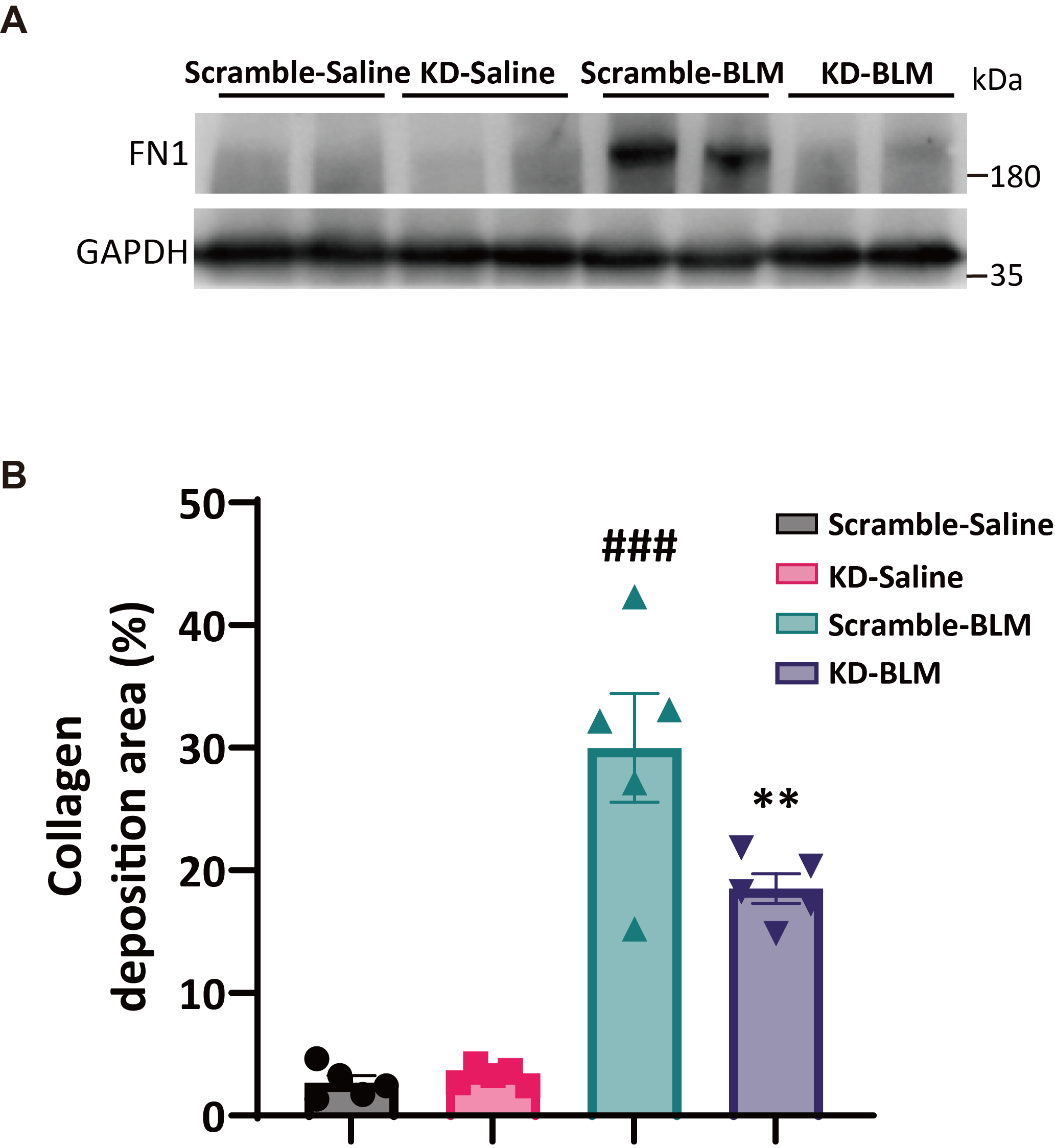


**Figure S10. RIPK3 knockdown in lung ameliorated BLM-induced pulmonary fibrosis.** **(A)** Western Blot analysis of FN1 in lung tissues. **(B)** The percentage of collagen deposition area via Masson’s trichome staining of lung tissues in Figure 7G. (B) Data show means ± SEM. Symbols on bar graphs represent individual mice. (B) one-way ANOVA with Dunnett’s multiple comparisons test were used. ^###^*P*<0.001, compared with the Scramble-Saline group. ^**^*P*<0.01 compared with the Scramble-BLM group.

**Table S1. Sequences of primers for real-time quantitative PCR and genotyping assays.**

**RT-qPCR**

| Gene | Forward | Reverse |
| --- | --- | --- |
| *β*-Actin | GTGACGTTGACATCCGTAAAGA | GCCGGACTCATCGTACTCC |
| *Ripk3* | GGCACCCTAGCGTACTTGG | GCTGTAGACATCACTCGCTTT |
| *Il-1β* | GAAATGCCACCTTTTGACAGTG | TGGATGCTCTCATCAGGACAG |
| *Il-6* | CTGCAAGAGACTTCCATCCAG | AGTGGTATAGACAGGTCTGTTGG |
| *Tnf-α* | CAGGCGGTGCCTATGTCTC | CGATCACCCCGAAGTTCAGTAG |
| *Col1a1* | GCTCCTCTTAGGGGCCACT | ATTGGGGACCCTTAGGCCAT |
| *Col3a1* | CTGTAACATGGAAACTGGGGAAA | CCATAGCTGAACTGAAAACCACC |
| *Fn1* | ATGTGGACCCCTCCTGATAGT | GCCCAGTGATTTCAGCAAAGG |
| *Arg1* | CTCCAAGCCAAAGTCCTTAGAG | GGAGCTGTCATTAGGGACATCA |
| *Fizz1* | CCAATCCAGCTAACTATCCCTCC | ACCCAGTAGCAGTCATCCCA |
| *Ym1* | CAGGTCTGGCAATTCTTCTGAA | GTCTTGCTCATGTGTGTAAGTGA |
| *Spp1* | ATCTCACCATTCGGATGAGTCT | TGTAGGGACGATTGGAGTGAAA |
| *Cx3cr1* | GAGTATGACGATTCTGCTGAGG | CAGACCGAACGTGAAGACGAG |
| *Col4a1* | CTGGCACAAAAGGGACGAG | ACGTGGCCGAGAATTTCACC |
| *Odc1* | GACGAGTTTGACTGCCACATC | CGCAACATAGAACGCATCCTT |
| *Sms* | CACAGCACGCTCGACTTCAA | TGCCATTCTTGTTCGTGTAAGTT |

**Genotyping**

| Mice Genotyping | Forward | Reverse |
| --- | --- | --- |
| Cx3CR1-Cre | GTGTTTTCTCCCGCTTGCTG | AGTGATGCTCTTGGGCTTCC |
|  | ACCAACAGGGTTCCAAGCAA | GTTGTTCAGCTTGCACCAGG |
| RIPK3^flox/flox^ | GCTACCTACACAGCTTGAACC | GTCATTGAGAACTTAGCAGGAG |
|  | CACGCCAAGGTTAGTCCATC | GCTGTGCCTCTTCAGAATTAGT |
| Rosa26^stop(flox/flox)Ripk3^ | TCAGATTCTTTTATAGGGGACACA | TAAAGGCCACTCAATGCTCACTAA |
|  | CAAGCACAGTTCGGCAGGGGTAGG | ACGCCGTAGGTCAGGGTGGTCA |

**Table S2.** **List of antibodies and reagents used in this study.**

**Antibodies**

| **Name** | **Source** | **Cat. Number** |
| --- | --- | --- |
| SMAD2/3 (D7G7) XP^®^ Rabbit mAb | Cell Signaling Technology | 8685 |
| Phospho-Smad2 (Ser465/467)/Smad3 (Ser423/425) (D27F4) Rabbit mAb | Cell Signaling Technology | 8828 |
| Fibronectin Rabbit pAb | Abclonal | A12932 |
| RIP3 (D4G2A) Rabbit mAb | Cell Signaling Technology | 95702 |
| p70 S6 Kinase Antibody | Cell Signaling Technology | 9202 |
| Phospho-p70 S6 Kinase (Thr421/Ser424) Rabbit mAb | Cell Signaling Technology | 9204 |
| Akt Rabbit Antibody | Cell Signaling Technology | 9272 |
| Phospho-Akt(Thr308)(244F9) Rabbit mAb | Cell Signaling Technology | 4056 |
| Phospho-Akt (Ser473) (D9E) XP^®^ Rabbit mAb | Cell Signaling Technology | 4060 |
| 4E-BP1 (53H11) Rabbit mAb | Cell Signaling Technology | 9644 |
| Phospho-4E-BP1 (Thr70) Rabbit mAb | Cell Signaling Technology | 9455 |
| Alexa Fluor^®^ 647 Anti-F4/80 antibody [F4/80] | Abcam | ab204467 |
| Osteopontin Polyclonal antibody | Proteintech | 22952-1-AP |
| HRP-conjugated monoclonal mouse anti-GAPDH | KangChen | KC-5G5 |
| FITC Goat anti-rabbit IgG (H+L) | Proteintech | SA00003-2 |
| Goat polyclonal Secondary Antibody to Rabbit IgG - H&L (Alexa Fluor^®^ 647) | Abcam | ab150079 |
| Ms Ly-6G/Ly-6C FITC RB6-8C5 | BD Pharmingen | 553127 |
| CD11b BV421 M1/70 | BD Pharmingen | 562605 |
| Ms CD45 BUV395 30-F11 | BD Pharmingen | 564279 |
| BV510 Rat Anti-Mouse Siglec-F(E50-2440) | BD Pharmingen | 740158 |
| PE hamster anti-mouse CD11c (HL3) | BD Pharmingen | 553802 |
| F4/80 Monoclonal Antibody (BM8), PerCP-Cyanine5.5 | Thermo Fisher Scientific | 45-4801-82 |
| eBioscience™ Fixable Viability Dye eFluor™ 780 | Thermo Fisher Scientific | 65-0865-18 |
| BV421 anti-mouse Cx3CR1 Antibody | Biolegend | 149023 |
| Brilliant Violet 421 rat anti-mouse I-A/I-E (M5/114) MHC-II | BD Pharmingen | 562564 |
| FITC Rat Anti-Mouse CD86 | BD Pharmingen | 561962 |
| APC anti-mouse F4/80 | Biolegend | 123116 |
| CD11b BUV395 M1/70 | BD Pharmingen | 563553 |
| CD206 (MMR) Monoclonal Antibody (MR6F3), APC | Thermo Fisher Scientific | 17-2061-82 |
| FITC conjugated anti-mouse B7-DC (CD273), PD-L2 (122) | Thermo Fisher Scientific | 11-9972-85 |
| CD16/CD32 Monoclonal Antibody | Thermo Fisher Scientific | 14-0161-86 |

**Reagents**

| **Name** | **Source** | **Cat. Number** |
| --- | --- | --- |
| Bleomycin Sulfate | TargetMol | T6116 |
| Zoletil-50 | Virbac |  |
| Trypsin-EDTA (0.25%), phenol red | Thermo Fisher Scientific | 25200056 |
| LongAmp Hot Start Taq 2× Master Mix | New England Biolabs | M0533S |
| 50×TAE buffer | Solarbio | T1060 |
| Premix Taq^TM^ DNA Polymerase | Takara | R004A |
| Proteinase K | Takara | 9034 |
| Trans2K Plus II DNA Marker | Transgen | BM121-01 |
| 6×DNA Loading Buffer | Transgen | GH101-01 |
| Agarose | Yeasen | 10208ES60 |
| 4SGelred, 10000X in water | Sangon Biotech | A616697 |
| Mouse IFN-gamma Recombinant Protein | Peprotech | 315-05 |
| Lipopolysaccharides from Escherichia coli O55:B5 | Sigma-Aldrich | L2880 |
| Mouse IL-4 Recombinant Protein | Peprotech | 214-14 |
| Mouse IL-13 Recombinant Protein | Peprotech | 210-13 |
| Mouse M-CSF Recombinant Protein | Peprotech | 315-02 |
| Mouse GM-CSF Recombinant Protein | Peprotech | 315-03 |
| Recombinant Mouse TGF-beta 1 Protein | R&D | 7666-MB |
| COLLAGENASE FROM CLOSTRIDIUM HISTOLYTICU | Sigma-Aldrich | C5138 |
| DISPASE II | Sigma-Aldrich | D4693 |
| DNASE I, GRADE II | Sigma-Aldrich | 10104159001 |
| BSA | Genebase |  |
| NON-Fat Powdered Milk | Sangon Biotech | A600669 |
| SuperBlock T20 (PBS) Blocking Buffer | Thermo Fisher Scientific | 37516 |
| Color PAGE Gel Rapid Preparation Kit | Epizyme Biotech | PG112 |
| SuperSignal™ West Pico PLUS | Thermo Fisher Scientific | 34580, 32109 |
| SDS Lysis Buffer | Beyotime | P0013G |
| Immunol Staining Fix Solution | Beyotime | P0098 |
| Immunohistochemical wash buffer | Beyotime | P0106C |
| Fluorescence mouting medium | Dako | S3023 |
| Immunol Staining Blocking Buffer | Beyotime | P0102 |
| Immunol Staining Primary Antibody Dilution Buffer | Beyotime | P0103 |
| Immunol Fluorescence Staining Secondary Antibody Dilution Buffer | Beyotime | P0108 |
| Trans-Blot Turbo 5x Transfer Buffer | Bio-rad | 10026938 |
| SDS-PAGE Sample Loading Buffer, 5X | Beyotime | P0015 |
| PageRuler Prestained Protein Ladder | Thermo Fisher Scientific | 26617 |
| Pierce™ Dilution-Free™ Rapid Gold BCA Protein Assay | Thermo Fisher Scientific | A55860 |
| RNAsimple Total RNA Kit | Tiangen | DP419 |
| DAPI Staining Solution | Beyotime | C0015 |
| Red Blood Cell Lysis Buffer | Beyotime | C3702 |
| DSP | Thermo Fisher Scientific | PG82081 |
| DEPC | Sigma-Aldrich | D5758 |
| dNTP | Thermo Fisher Scientific | R0192 |
| T4 DNA Ligase | New England Biolabs | M0202 |
| Protease | Qiagen | 19155 |
| Phanta super-fidelity DNA polymerase | Vazyme | P505 |
| VAHTS beads | Vazyme | N411 |
| Gel Extraction Kit | New England Biolabs | T1120 |
| EasySep^TM^ Mouse F4/80 Positive Selection Kit | Stem cell | 100-0659 |
| Hydroxyproline assay kit | Nanjing Jiancheng | A030-2-1 |
| Hifair^®^ AdvanceFast One-step RT-gDNA Digestion SuperMix for qPCR | Yeasen | 11151ES60 |
| Hieff^®^ qPCR SYBR^®^ Green Master Mix （Low Rox Plus） | Yeasen | 11202ES08 |
| IC Fixation Buffer | Thermo Fisher Scientific | 00-8222-49 |
| Ms TNF Pure G281-2626 | BD Biosciences | 551225 |
| Ms TNF Biotin MP6-XT3 | BD Biosciences | 554415 |
| Ms IL-6 Pure MP5-20F3 | BD Biosciences | 554400 |
| Ms IL-6 Biotin MP5-32C11 | BD Biosciences | 554402 |
| Ms IL-12 (p40/p70) Pure C15.6 | BD Biosciences | 551219 |
| Ms IL-12 p40/p70 Biotin C17.8 | BD Biosciences | 554476 |
| Streptavidin-HRP | BD Biosciences | 554066 |
| TMB Solution (1×) | Thermo Fisher Scientific | 00-4201-56 |
| Ms TNF Recom | BD Biosciences | 554589 |
| Ms IL-6 Recom | BD Biosciences | 554582 |
| Ms IL-12 p40 Recom | BD Biosciences | 554594 |
| RPMI 1640 medium | Gibco | C22400500BT |
| DMEM medium | Gibco | C11995500BT |
| IMDM medium | Gibco | C12440500BT |
| FBS | HyClone |  |
| EasySep Mouse F4/80 Pos Sel Kit | Stem Cell | 100-0659 |
| Spermidine | J&K | 184628 |
| Spermine tetrahydrochloride | J&K | 900515 |
| Penicillin-Streptomycin | Yeasen | 60162ES76 |
| Nec-1s | MedChemExpress | HY-14622A |
| GSK’872 | MedChemExpress | HY-101872 |
| Omipalisib | MedChemExpress | HY-10297 |

**Reference:**

1. Ruscitti C, Abinet J, Maréchal P, Meunier M, de Meeûs C, Vanneste D, et al. Recruited atypical Ly6G(+) macrophages license alveolar regeneration after lung injury. Sci Immunol. 2024;9(98):eado1227.

2. Morse C, Tabib T, Sembrat J, Buschur KL, Bittar HT, Valenzi E, et al. Proliferating SPP1/MERTK-expressing macrophages in idiopathic pulmonary fibrosis. Eur Respir J. 2019;54(2):1802441.
